# Integration, Disintegration, and Self-Similarity: Characterizing the Scales of Shape Variation in Landmark Data

**DOI:** 10.1007/s11692-015-9317-8

**Published:** 2015-04-19

**Authors:** Fred L. Bookstein

**Affiliations:** Faculty of Life Sciences, University of Vienna, Vienna, Austria; Department of Statistics, University of Washington, Seattle, WA USA

## Abstract

The biologist examining samples of multicellular organisms in anatomical detail must already have an intuitive concept of morphological integration. But quantifying that intuition has always been fraught with difficulties and paradoxes, especially for the anatomically labelled Cartesian coordinate data that drive today’s toolkits of geometric morphometrics. Covariance analyses of interpoint distances, such as the Olson–Miller factor approach of the 1950’s, cannot validly be extended to handle the spatial structure of complete morphometric descriptions; neither can analyses of shape coordinates that ignore the mean form. This paper introduces a formal parametric quantification of integration by analogy with how time series are approached in modern paleobiology. Over there, a finding of trend falls under one tail of a distribution for which stasis comprises the other tail. The null hypothesis separating these two classes of finding is the random walks, which are *self-similar,* meaning that they show no interpretable structure at *any* temporal scale. Trend and stasis are the two contrasting ways of deviating from this null. The present manuscript introduces an analogous maneuver for the spatial aspects of ontogenetic or phylogenetic organismal studies: a subspace within the space of shape covariance structures for which the standard isotropic (Procrustes) model lies at one extreme of a characteristic parameter and the strongest growth-gradient models at the other. In-between lies the suggested new construct, the *spatially self-similar processes* that can be generated within the standard morphometric toolkit by a startlingly simple algebraic manipulation of partial warp scores. In this view, integration and “disintegration” as in the Procrustes model are two modes of organismal variation according to which morphometric data can deviate from this common null, which, as in the temporal domain, is formally featureless, incapable of supporting any summary beyond a single parameter for amplitude. In practice the classification can proceed by examining the regression coefficient for log partial warp variance against log bending energy in the standard thin-plate spline setup. The self-similarity model, for which the regression slope is precisely $-1,$ corresponds well to the background against which the evolutionist’s or systematist’s a-priori notion of “local shape features” can be delineated. Integration as detected by the regression slope can be visualized by the first relative intrinsic warp (first relative eigenvector of the nonaffine part of a shape coordinate configuration with respect to bending energy) and may be summarized by the corresponding quadratic growth gradient. The paper begins with a seemingly innocent toy example, uncovers an unexpected invariance as an example of the general manipulation proposed, then applies the new modeling tactic to three data sets from the existing morphometric literature. Conclusions follow regarding findings and methodology alike.

## Prologue

Contemporary morphometrics arose as a subdiscipline of biometrics, assembled mostly from borrowed tools (shape theory, multivariate statistics, analytic geometry, interpolation theory, medical image analysis), that turns out to have applications all across the quantitative organismal biosciences. Some branches of applied mathematics and biomathematics, like shape theory, were mined very wisely and well in the course of building this toolkit; other branches, like multivariate statistical analysis, perhaps not so wisely. This article, which takes the form of an extended essay, introduces a new parameter for the *scaling* of shape variation, together with an exegesis of the shape patterns expected from shape data when that parameter takes on a particularly interesting nonzero value.

The new approach to scaling for landmark data was first hinted at in technical papers about the thin-plate spline not intended to be read by biologists. But if whole-organism developmental mechanics, functional morphology, and evolutionary biology are to continue fruitfully to exploit the very convenient and suggestive formalism of landmark data, the scaling praxis must now be revisited and revisualized in the biologist’s own diagrammatic language. The parameterization I am suggesting here will have major implications for a specific aspect of multivariate description, the elucidation of *integration,* that is presently in an incoherent state, however intuitive its current tools may seem. The proposal is to render this intuition coherent by radically rethinking the notion of a “null model” for integration—what it means to *not* be integrated—so as no longer to require that covariances of shape coordinates be centered around zero. The new construct is intended to replace a motley of classical notions of integration, usually based on examination of covariance structures without reference to the corresponding average shapes, that cannot be successfully translated into the landmark-based setting.

It is not that the abstractions that follow here are wholly unfamiliar to the practicing biologist. Anyone examining samples of multicellular organisms in anatomical detail must already have an intuitive concept of morphological integration. That same practicing biologist knows perfectly well that some quantifiable features of organismal form-comparisons over ontogeny or phylogeny are measured at large scale, using rulers calibrated in centimeters and commensurately large protractors, while other features are measured at small scale, using miniature rulers or tiny protractors. But quantifying that intuition has always been fraught with difficulties and paradoxes, and particularly so for the anatomically labelled Cartesian coordinate data that drive today’s toolkits of geometric morphometrics. Covariance analyses of interpoint distances, such as the Olson–Miller factor approach of the 1950’s, cannot validly be extended to handle the spatial structure of morphometric descriptors; neither can analyses of shape coordinates that ignore the mean form. I will touch on these and other paradoxes and infelicities of today’s typical approaches at various points in the sequel.

**Fig. 1:**
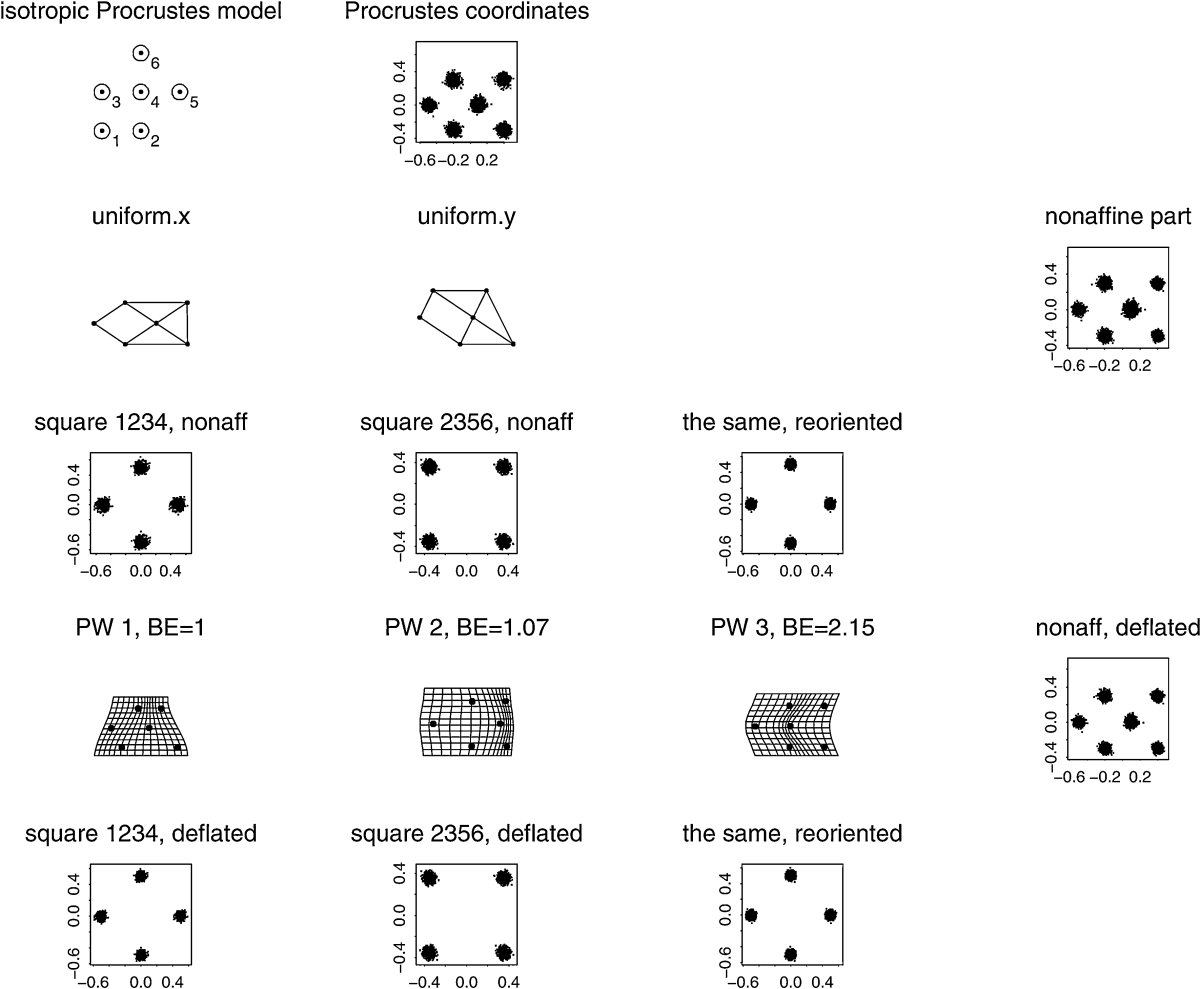
Explicit construction of the self-similar domain of variation for a toy data set of six landmarks (a simulation of the offset isotropic Mardia–Dryden model on the loci in the upper left panel). The variances of the two distributions of square shape examined in the last row of the figure are the same even though their geometric scales differ by a factor of $\sqrt{2}$. The deflation maneuver that is the subject of Sect. "A Theorem with Its Corollary Algorithms" of this paper protects us from being misled into thinking that the large square was an intrinsically less variable sort of “feature” just because of its large size. The detailed descriptions of the panels in this figure are together too lengthy to be laid out in this caption; please see the Prologue

But this Prologue is not intended mainly as a review of those difficulties. Instead its diagrams, all tumbled together in the single multipanel Fig. 1, combine the standard tools of geometric morphometrics in a new way in order to reveal a surprising *invariant* aspect of the Procrustes geometry hidden in a convenient toy data set. The Prologue is followed by a more conventional introduction reviewing the literature pertinent to the new tool, including references to an earlier, more mathematical literature arguing, albeit implicitly, that the “surprise” must in fact be ubiquitous wherever the thin-plate spline approach is combined with a certain very specific simulated Procrustes distribution of shape coordinates. From this ubiquity follows the principal conclusion of the paper: this particular subclass of Procrustes shape coordinate distributions should be embraced as the proper “null model” for studies of integration. The model is entirely different from the models of uncorrelated variation in that it is conditioned on the exact details of the spacing of the points in an average landmark configuration in such a way as to avoid privileging any particular geometrical scale of features over any other scale. I proceed with a thorough explication of the detailed algebra of this approach, including the formulas that should allow any morphometrically adept reader to duplicate my calculations; then three separate worked examples involving previously published data sets; and finally a closing Discussion.

By way of setting the scene for the maneuvers to follow, the reader’s attention is called to the 15 panels of Fig. 1.

At upper left is a schematic of the conventional *offset isotropic Gaussian model* for shape variation around an average. Shapes will vary around a mean form comprising the six points numbered as shown, evidently derived from a square grid such as is found on ordinary graph paper. The circles around the six points are drawn at 2 standard deviations of the underlying circular normal (Gaussian) variation assigned to every landmark independently. There results, of course, the familiar *offset isotropic Mardia–Dryden model* for the variation of the corresponding *shape* of the landmark configuration (Dryden and Mardia 1998). To its right in this upper row is a simulation of 1000 draws from this shape distribution, as presented in the conventional *Procrustes shape coordinate plot* after centering, scale, and orientation have all been standardized. The bilateral symmetry of this configuration across its horizontal axis is visually pleasing but actually has no role to play in the argument here. For small standard deviations, when shape is represented by a suitable projection of these twelve coordinates ($x$ and $y$ for each of the six points) it is well-known that the representation lies in a linear subspace of dimension eight.

The second row of Fig. 1 shows one particular follow-up manipulation of these Procrustes coordinates, the separation of two of the eight dimensions from the other six. This separation is nothing new. It was already diagrammed in Bookstein (1991), and was most recently formalized in matrix notation on page 418 of Bookstein (2014). The dimensions we seek to separate out are the two dimensions of the *uniform* or *affine transformations*, those that leave straight lines straight and midpoints midpoints. These transformations are spanned by the two exemplars at left in this row of the figure, which correspond to projections along rows 1 and 2, respectively, of the matrix written out in full in Eq. (7) below. As drawn, neither of these changes is actually within the Procrustes shape space itself—I omitted the rotation and rescaling steps—but it is easier to appreciate the construction to follow if the forms are left in this mixed coordinate system. The uniform transformations here will be highlighted below as the algebraically simplest characterization of the *totally integrated transformations.* In the representation as maps here, they are characterized by having the same affine derivative at every point of the organism. In other versions it will be the second derivative that is modeled as constant in this global (organism-wide) way, so as to include the homogeneous growth-gradients as well.

Still in the second row, at far right is the scatterplot of Procrustes shape coordinates after these two uniform (affine) dimensions have been partialled out of the simulation. In terms of the original shape space, we have removed two dimensions out of the eight that were needed to characterize the original spherical shape variation. What remains has a different covariance structure than the original shape coordinates—in particular, its rank is now six, not eight—and it has a trace (sum of the variances of all the coordinates here) that is just $6/8=75\%$ of what the trace was before.

Turn now to the third row. Here I have selected two different subconfigurations of the six-landmark scheme that have the same expected shape (an exact square, according to the mean form) but different sizes. There is a smaller square, on the landmarks numbered 1, 2, 3, and 4 at upper left, and also a larger square (landmarks 2, 3, 5, 6) on the diagonal of the smaller square. Thus the two squares differ in scale by a factor of exactly $\sqrt{2}$. We are interested in the nonuniform variation of these two subconfigurations of landmarks—the extent to which both can be characterized by local features (which, for this mean configuration, are the “square-to-kite” and “square-to-trapezoid” processes that concern us in more detail in Fig. 8 below). Each of these is a descriptor space of exactly two dimensions (as the nonaffine space for any starting set of four landmarks would be). At far left in this row is the plot corresponding to that in the second row for just the smaller square, landmarks 1, 2, 3, 4. Inspection of a copy printed at much larger scale reveals that the distributions at ends of a diagonal are identical and those at opposite ends of an edge are opposites. Furthermore, the variation is obviously circular in the plane of the diagram. Then the net extent of variability can be summarized by the variance of any single Cartesian coordinate at any landmark. That variance turns out to be 0.001247 (in Procrustes units).

To its right is the same construction on the larger square (landmarks 2, 3, 5, 6). Again there are only two available dimensions of shape variation—ends of diagonals have the same pattern, ends of edges exactly opposite patterns. The scale of the Procrustes shape coordinate plot has changed only because of the orientation of the square upon the original form. When that is adjusted (see the rightmost plot in this row) we can see that the Procrustes variance of these shapes is much smaller for this larger square than for the smaller square 1, 2, 3, 4. We compute it as 0.000662, which, for this sample of 1000 simulations, is indistinguishable from precisely half the nonaffine shape variance of the smaller square. In other words, the variance of features of squares varies as the inverse of the area of the square: ${1\over 2}=(1/\sqrt{2})^2.$ This will prove unhelpful when we are trying to interpret principal components of shape.

Consider now the grids in the fourth row of this figure. These are all of the *principal warps* of the configuration of six landmarks here, each one drawn at the same amplitude (0.25 Procrustes units) at the mean form along the direction of that axis of bilateral symmetry ($x$-axis in the diagram). Above each is written its specific *bending energy,* the net integral of summed squared second derivatives of the corresponding spline map taken over the whole picture plane, as in Eq. (5) below. It is known that these energies can be derived as eigenvalues of the *bending-energy matrix* for this mean landmark configuration, the formalism set out in detail in the exposition below. As printed on the figure, these eigenvalues are proportional to 1:1.07:2.15. One could draw each of these principal warps as well in the application to the other Cartesian coordinate of this situation, the $y$-coordinate instead of the $x$-coordinate, or to their combination as real and imaginary components of the same maneuver, the construction of the *partial warp scores,* which are now in the complex $(x,y)$ plane. Because the principal warps are functions only of the mean configuration, and because they are perpendicular in shape space, they constitute a statistically arbitrary orthogonal rotation of the original Procrustes variation, which is spherical in all eight of its dimensions. The uniform terms and the partial warps are four orthogonal two-dimensional subspaces of this eight-dimensional space, and so the simulation should show the same shape variances for each of the four. In fact we get variances of 0.00337, 0.00359, 0.00350, and 0.00344, which do not meaningfully differ—in this most familiar of the Procrustes shape coordinate models there is no spatially differentiated pattern to be found. Thus this data has no spatial structure. Rather, it is, using the neologism to be introduced below, totally “disintegrated,” which is to say, incompatible with life.

Taking all this for granted, we can produce a *deflation* of the observed Procrustes variation—in reality one route to the production of a relative eigenanalysis (see below)—by reducing the variance of each partial warp by a factor proportional to its specific bending energy. By doing so we will make possible (though it will not be demonstrated until Sect. "Visualizing Integration: Three Examples" below) the construction of a new set of principal components that are diagonalized not in terms of Procrustes distance but in terms of bending energy. Since this quantity is zero for the uniform transformations, the calculation must be restricted to the nonaffine subspace of shape, the subspace we are working in here. There results the new “deflated” scatter of Procrustes coordinates shown at the far right in this fourth row, directly under the original, undeflated version in the second row. Plotted in this fashion, it is not at all obvious what has changed.

What has changed over the deflation, in fact, is the biologist’s language of pattern analysis for these coordinates. To see this, examine the scatters in the last row of the figure, each of which is aligned with one of the scatters in the third row. There they dealt with variations of nonaffine shapes of subsquares in the Procrustes coordinates, and we saw that the variance was inverse to the area of the squares. Here in the fifth row, by contrast, *the variance of the nonaffine component of the shape of these perturbations of squares is ***independent of scale!** The visual extent of the little circles in the nonaffine scatters for the 1, 2, 3, 4 square, far left, and for the 2, 3, 5, 6 square, far right, are *nearly identical.* In fact the variances of the two are 0.000629 and 0.000663. Again these variances do not differ; but this time they are variances of the *same* shape feature as it would be reported at two *different* spatial scales.

By deflating each dimension of nonaffine shape space by the bending energy of its principal warp, then, we have produced a shape distribution for which the original equality of variances of equally important potential features is *replicated* at this particular contrast of scales. The distribution of nonaffine shapes of square subconfigurations of landmarks, in other words, is now *self-similar,* the same at *every* available geometric scale (there are only two available in this example). This will prove to be the case for *every* landmark or semilandmark configuration. Of even greater importance for our applications is the obverse of this proposition: what we intend when we report a specific “shape feature” is to be construed as a feature of the *deviation of shape variability from this model.* Not the principal components with respect to Procrustes distance (the “relative warps” appearing in the overwhelming majority of papers that use geometric morphometrics to analyze real organismal data sets) but the principal components with respect to *bending energy* constitute the tool we should be using to search for meaningful characters across the full range of scales available to our characterizations of living or dead organisms.

## Introduction

**Fig. 2:**
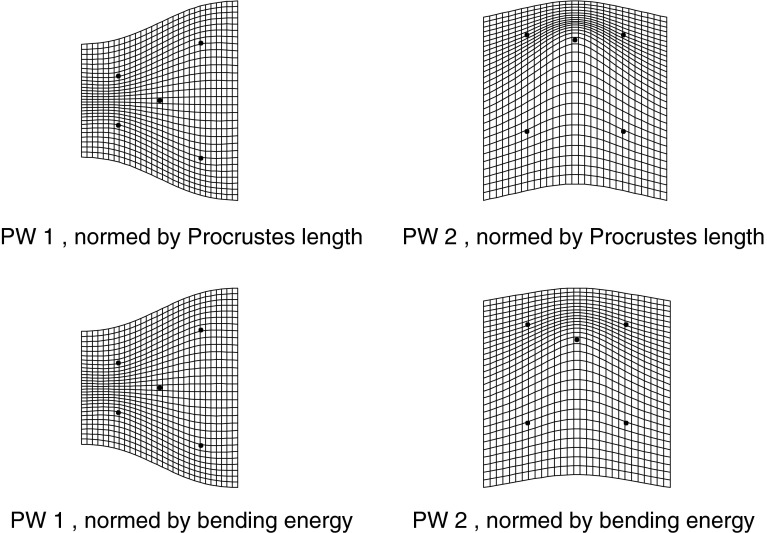
The two nontrivial principal warps for a quincunx of landmarks (the shape of the five-spot of a die), as represented by thin-plate splines. Above, normed to the same Procrustes length; below, to the same bending energy, which deflates the more bent principal warp (*right column*) by a factor of $\sqrt{.6}$. After the deflation, the visual density of grid lines is much more nearly equal at their loci of greatest density (*left column*, *center left*; *right column*, *upper center*). Informally, bending energy is the integrated squared rate of change of this pattern of densities when it is drawn all the way out to infinity in all directions

How is it that deflation by bending energy serves to equalize phenomena at different scales? Let’s look at an even simpler example, the bending energy for a quincunx of landmarks (the pattern of dots on the side of a die that has five of them). From the formula to follow in Sect. "A Theorem with Its Corollary Algorithms", this will prove to be proportional to the quadratic form

$$\begin{aligned} B_Q=\left( \begin{array}{ccccc} 2&-1&2&-1&-2\\ -1&2&-1&2&-2\\ 2&-1&2&-1&-2\\ -1&2&-1&2&-2\\ -2&-2&-2&-2&8\\ \end{array}\right) , \end{aligned}$$

for which the only eigenvectors of nonzero eigenvalue are the patterns $$W_1=(1,-1,1,-1,0)$$ and $$W_2=(1,1,1,1,-4). $$ (The central element of the quincunx corresponds to row or column 5 in these expressions, and for $$W_1$$ the other landmarks have been numbered consecutively around the outline.) After these vectors are normalized to unit length we have $$W_1^tB_QW_1=6, W_2^tB_QW_2=10.$$ The two specific bending energies are thus in the ratio of 3 to 5, as shown in Fig. 2.

This diagram is intended to clarify the role of the bending energy in rendering further comparisons relatively scale-free. Look at the grid spacings where they are densest: the gradients away from these loci contribute the most to the bending energy integral. Informally, what we are doing is (approximately) normalizing to that densest spacing of the lines (see the figure). These spacings convert to potential shape features roughly as ratios to lengths that are unchanging: for $W_1,$ the ratio of the length of the left edge, or the right edge, to the width of the quincunx on its page; for $W_2,$ the ratio of height (in the page’s vertical) of the upper triangle of landmarks, or the lower, to this same width. The effect of the switch to the bending-energy norm is to render the maximum (densest) spacing roughly equal between the two dimensions of variation, and hence to calibrate the *intensity* of a shape feature, the integrated squared rate of change of densities like these, in a way that is relatively independent of its geometric scale (which is considerably smaller for the second principal warp than for the first). The situation is the same for the general landmark configuration: normalization by bending energy reduces all changes in the nonaffine space, regardless of approximate geometric extent, to the same currency of derivatives of this contour density, squared and then integrated over the picture.

Such a procedure strikingly resembles a technique that has been known for over a hundred years to apply in the temporal domain: the normalization of random walks and diffusions such as Brownian motion by the square root of time. In the technical jargon, both are *Gaussian increment processes.* In other words, the resemblance is more than mere analogy: our bending-energy maneuver is actually a strict mathematical generalization of the Brownian motion case. (See Mardia et al. 2006, especially sections 2.1–2.3.) Perrin (1913/1923) received the Nobel Prize in Physics for demonstrating the validity of this self-similar scaling formalism as it applies to real Brownian motion on the Einstein model (see Bookstein 2014, Section E4.3.2). In that physical setting, the variance of a Brownian motion can be shown to vary linearly in elapsed time.

From the fact (or, rather, the theorem) of this temporal scaling, it proved possible to convert the study of paleontological time series from the relatively fruitless consideration of models against a null of *no change* to a much more fruitful null model, the temporal integration of *independent increments* corresponding to the neutral model of phenotypic evolution (see, e.g., Nei 2007). The simple suggestion of computing a scaling dimension for the extent of maximum change, in particular, led, over the course of a quarter of a century, to a great radiation of methods for the analysis of these series. As presented in Bookstein (1987, 1988) for univariate series and Bookstein (2013) for multivariate series such as sequences of shapes, the role of random walk is as a null model affording access to interpretable biological phenomena in *both* of its tail directions. For series that are hyp*er*variant with respect to this linear model, the rejection of the null is an assertion of trend; for series that are instead hyp*o*variant, rejection entails the contrary finding, stasis. Recognizing the manner of scaling of random walks with time induced a relocation of the null hypothesis for evolutionary series from constancy of a mean (stasis) to neutral drift. The vocabulary by which these time series could be discussed in biologically meaningful terms, along with their causes or effects, was correspondingly enriched.

**Fig. 3:**
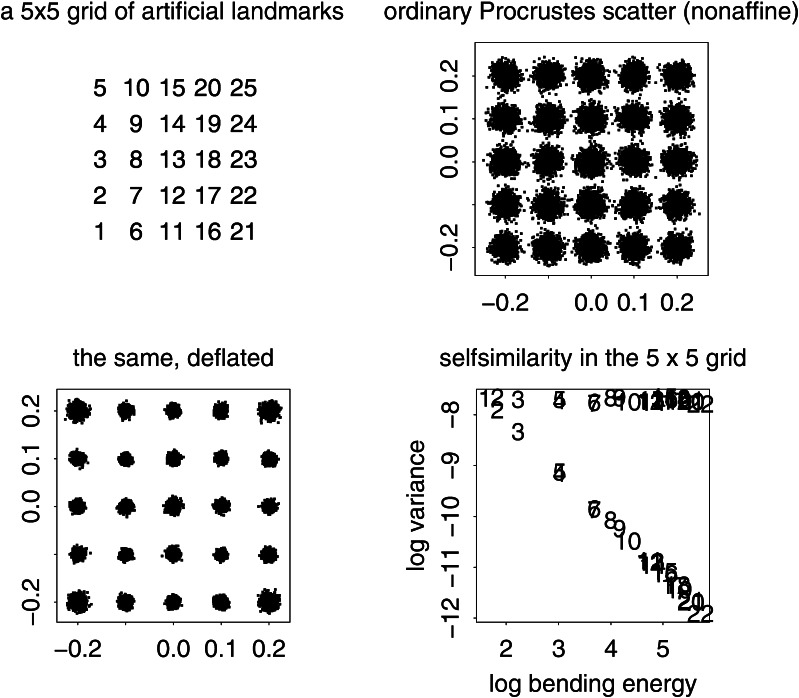
The isotropic offset Gaussian distribution for a $5\times 5$ square grid of artificial landmarks. The standard deviation of the isotropic offset Gaussian process was set to 0.15 of the unit cell spacing. (*upper left*) The landmarks, numbered for use in Fig. 5. (*upper right*) The Procrustes shape distribution after the two-dimensional affine term has been projected out. (*lower left*) The bending-deflated version. (*lower right*) Confirmation of the self-scaling claim in the text: the relation between feature scale (specific bending energy) and feature variance is precisely loglinear with a slope of $-1$ for the 22 partial warps of this artificial configuration after the deflation. Upper line: original variances by partial warp, slope $\sim 0.$ Lower line: variances after deflation, slope $\sim -1,$ to be confirmed by the explicit analyses for squares in Fig. 5

**Fig. 4:**
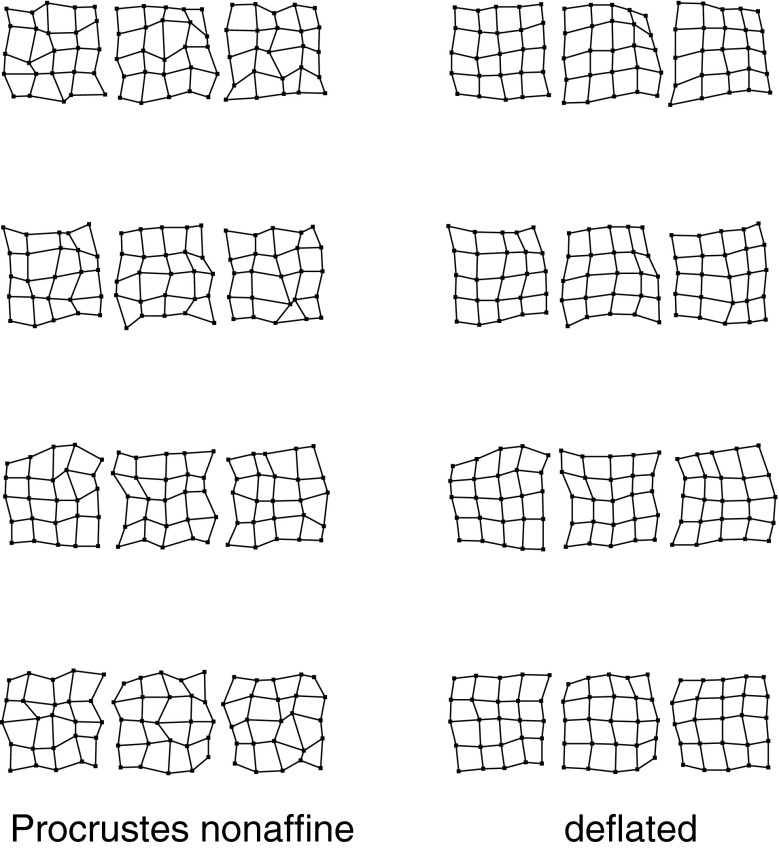
(*left*) A selection of grids near the 95th percentile of Procrustes distance from the distribution in the upper right panel of Fig. 3. These grids do not suggest any biologically meaningful interpretations—they are too disorganized. (*right*) The same for the deflated versions of the same grids (that is, the same specimens, but now drawing shape coordinates from the distribution at lower left in Fig. 3). The majority of these now suggest biological interpretability in terms of a small number of features at a discrete set of spatial scales

**Fig. 5:**
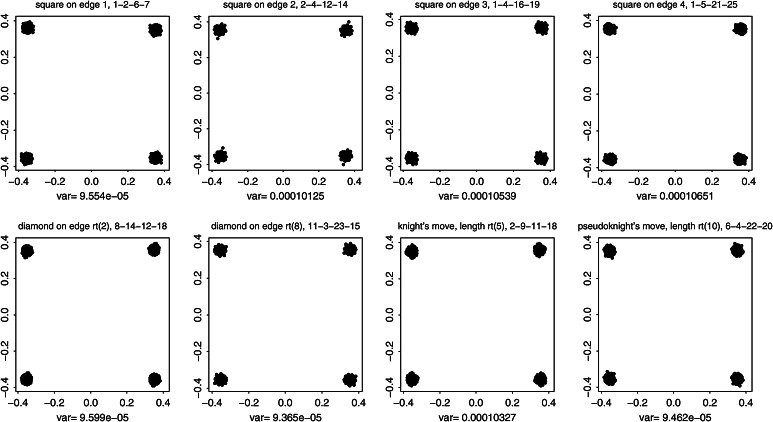
A more accessible demonstration that the bending-deflated shape distribution at *lower left* in Fig. 3 is self-similar. All integers in panel titles correspond to the landmark numbering scheme at *upper left* in Fig. 3. (*top*) Nonaffine shape of selected squares having edge lengths 1, 2, 3, or 4 unit cells with edges horizontal and vertical. (*bottom*) The same for squares aligned with the grid diagonal, having edge lengths $\sqrt{2}$ and $\sqrt{8}$, and for knight’s-move squares with subscript shifts like $(2,1)$, hence edge length $\sqrt{5}$, or $(3,1)$, of edge length $\sqrt{10}$. All of these shape distributions are the same. In other words, the deflation of bending energy corresponds to a self-similar model of shape variation against which it is reasonable to test empirically encountered data for the existence of patterns that deviate from the model. This and every other explicit comparison of distributions over identical subconfigurations are guaranteed invariant by the slope of $-1$ for the plot at *lower right* in Fig. 3

The present paper intends just such a recentering for the complementary domain of spatial variation (and, by extension, their joint combination in the spatiotemporal processes that are of central interest in the evo-devo sciences and in phylogenetic inference). The difference between the two approaches to a null model is usually more dramatic than what was demonstrated in the Prologue. For instance, from a set of (artificial) landmarks in a $5 \times 5$ grid, we can generate precisely 50 different squares that vary by size, grid position, and orientation. In the isotropic Procrustes model, the nonaffine shape variance of these squares itself varies strongly by size and to some extent by position and orientation as well. After the deflation by bending energy, though, they all show exactly the same distribution of nonaffine shape. Figure 3 numbers the landmarks and displays the basic Procrustes and bending-deflated scatters. The concluding panel shows the proportionality of variance after deflation to bending energy in the form of the log-log plot with slope $-1$ in order to anticipate the findings in two of the empirical examples in Sect. "Visualizing Integration: Three Examples", which extract other slopes for this same plot in realistic settings. It is this slope that stands for the actual parameter of integration when integration is actually found to be a meaningful partial description of a data set. Figure 4 collects examples of forms over a narrow range of Procrustes distances, showing how biologically uninterpretable the majority of such shape dimensions would be, and then the corresponding bending-deflated grids, which would be much more suggestive of interpretable biological patterns were they to have arisen in real data analyses. Figure 5 confirms that in the deflated version of the isotropic Procrustes distribution, the nonaffine shape variation of any square highlighted within this grid is not dependent on the size, position, or orientation of that square upon the mean landmark configuration of Fig. 3. It is quite startling that such a distribution of multiple shape coordinates should exist at all, let alone that it can be generated from the standard Procrustes shape space by such a simple manipulation.

What makes the grids at the right in Fig. 4 interpretable is the possibility of reporting via a short list of superposed large-scale and small-scale patterns. The large scale patterns, we will see in our Vilmann rodent skull example below, are a geometrization of the growth-gradients introduced by Julian Huxley in his *Problems of Relative Growth* of 1932 as previously formalized for Bookstein coordinates (two-point shape coordinates) in Bookstein (1991). The small-scale features can be considered as generalizations of the second principal warp for the quincunx already shown in Fig. 2: the relocation of a single landmark with respect to the location it would be assigned by the larger-scale transformation of some cell of the landmark grid within which it finds itself. That is, the grids at right in Fig. 4, which constitute a sample from the bending-deflated distribution, appear to be hierarchical in their features, whereas those at the left in the same figure, derived from the original Procrustes shape distribution, all exemplify the pattern that will be called “disintegrated” below. 1

The technique recommended in this paper will combine two aspects of any actual Procrustes data set, the covariances and the mean—the covariance patterns of shape coordinates (normalized distances from an orthogonal pair of lines through the centroid) and the spatial disposition of the relative positional shifts that account for those patterns—that have hitherto been kept analytically separate in our literature, to the great disadvantage of that literature. Whenever individual landmarks contribute to more than one distance, there is no obvious extension of either the Olson and Miller (1958) approach to “morphological integration” or any other popular covariance-based style of factor analysis of multiple measured distances that can properly take into account the spatial arrangements of those distances. The analysis of deformations by relative warps, on the other hand, inappropriately privileges end-to-end gradients over more local shape phenomena even when the local phenomena involve shape changes at larger ratios or otherwise of larger magnitude when assessed appropriately locally. All the existing protocols known to me for “testing” models of this fused domain inappropriately compare the observed patterns to models of noncorrelation rather than to the models of spatially cumulative random fields that clarified the corresponding literature for time series. The technique of bending-energy-based deflation on which this essay focuses represents the extension of the time series analysis (scaling of variance to linear time, the expectation on a random walk) to the two-dimensional or three-dimensional context of landmarks dispersed in multiple *spatial* dimensions.

**Fig. 6:**
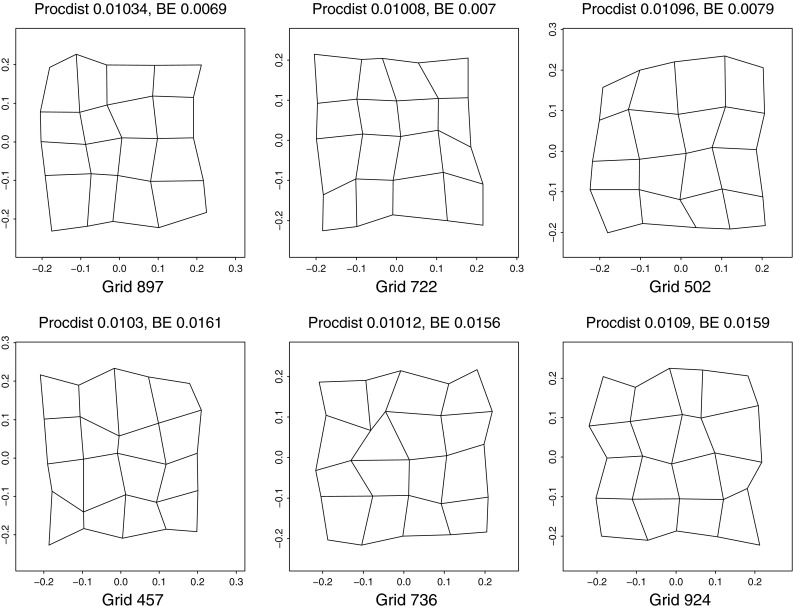
A selection of six grids drawn from the Procrustes simulation in Fig. 3 that all have approximately the same Procrustes distance from the mean (about 0.1). (*top*) The three of lowest bending energy, relatively more consistent with the biologist’s intuition of what an integrated feature can be expected to look like. (*bottom*) The three of highest bending energy, less biologically suggestive in the same sense (in other words, more difficult to reduce to ordinary verbal summaries). The deflated shape distribution of the proposal here is in effect the substantial overweighting of distributions of the upper type with respect to those of the lower type, and the less localized the bending, the greater the overweighting

The distinction I am making here can be clarified by comparing two sets of $5\times 5$ grid transformations that all have roughly the same Procrustes amplitude. Figure 6 shows three grids selected from the 1000 involved in Fig. 3 that all have Procrustes distance about 0.1 (before deflation) from the starting square configuration. These were selected from a set of 92 at distances between 0.10 and 0.11 to exemplify two extremes. At the top are the three grids that have the lowest bending energy—the likeliest to turn up from our deflated isotropic Procrustes distribution. These seem relatively legible in terms of their reportable features, for instance, the relative enlargement of the upper right quadrant in grid 502 or the U-shaped dilation of the vertical at left in grid 897. The grids of highest bending energy, by contrast, show a wholly disordered pattern of perturbations not consistent with any suggestive verbal summary. If the examples of low bending energy in the top row appear to be integrated, with features that are positively correlated from cell to neighboring cell, then those in the bottom row surely should be considered disintegrated, lacking in any such features. For more discussion along these lines, see Bookstein (2015).

**Fig. 7:**
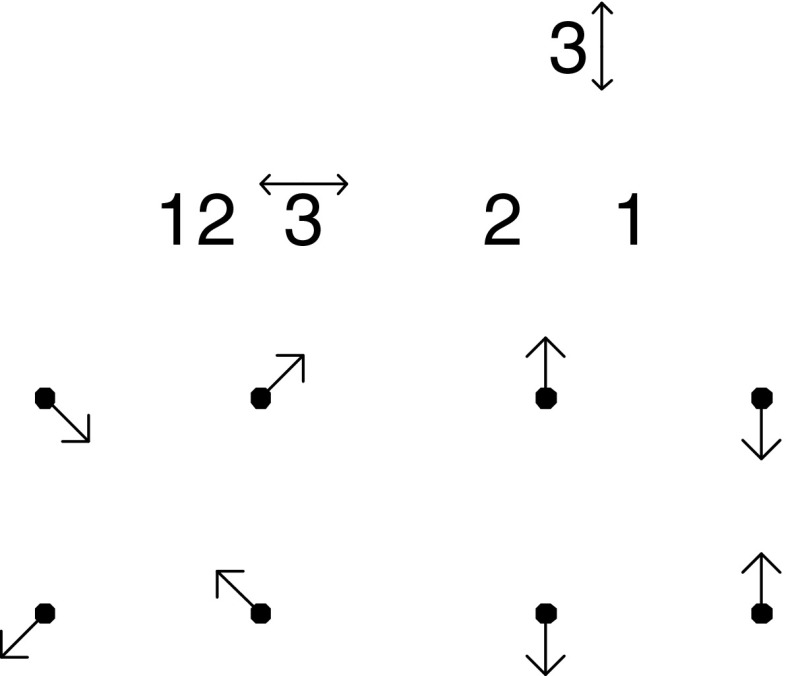
Two simple demonstrations of the fundamental paradox of interpoint distance analyses: no covariance pattern can be interpreted unambiguously unless the mean landmark configuration is an explicit component of the pattern analysis. In every panel, the arrows indicate the loadings of a factor that changes only the indicated coordinate(s) while leaving all others invariant. (*top*) Two triangles of landmarks having the same covariance matrix of all pairwise distances (see text) that nevertheless correspond to wholly different biological interpretations. (*bottom*) Two instances of the same covariance pattern (again see text) for two different *numberings* of the six pairwise distances among the four landmarks of the same mean configuration, again corresponding to entirely different biological interpretations, inasmuch as the segments corresponding to the distances that increase or decrease relatively fastest intersect in the scheme at *lower left* but are parallel in the scheme at *lower right*

I mentioned in the Prologue that the customary approaches to morphological integration based on correlations among multiple dimensions of descriptors do not suit our formalisms of Procrustes shape coordinates; it is time that I justified that claim. Figure 7 conveys two easily summarized paradoxes in this covariance-based morphometrics of distance data in order to conclude that no covariance pattern can be interpreted unambiguously unless the mean landmark configuration is an explicit component of the pattern analysis. The two triangles shown in the upper row are characterized by the same covariance structure

$$\begin{aligned} \sigma ^2{\left( \begin{array}{ccc} 0&{}0&{}0\\ 0&{}1&{}1\\ 0&{}1&{}1\\ \end{array} \right) } \end{aligned}$$

for the full set of three pairwise distances, where $\sigma$ is any sufficiently small quantity and the distances are taken in the order 12, 13, 23. (That points 1 and 2 are at an invariant distance suggests that all three points might have been represented by their Bookstein coordinates at the outset of the example.) But the two descriptions of the “same” pattern are nevertheless remarkably different when considered as evidence of biological processes. On the left, landmark 3 is restricted to the line through landmarks 1 and 2. On the right, landmark 3 is restricted to their perpendicular bisector, which makes as large an angle (90°) with the collinearity constraint as it possibly could. If we add a parameter $f$ for the failure of this canalization—the signed variation of point 3 away from the line along which it was supposed to be canalized—then the rate at which the variance of the difference of distances $d_{13}-d_{23}$ rises, and hence cov$(d_{13},d_{23})$ falls, is at least eightfold greater as a function of var$(f)$ for the second configuration than it is for the first.

In that example, the mean landmark configurations had different shapes. Yet even when we restrict our attention to comparisons having the same average shape, severe anomalies of interpretation can arise. In the lower row of Fig. 7 are two representations of a different covariance matrix

$$\begin{aligned} \sigma ^2\left( \begin{array}{cccccc} 1&{}-1&{}0&{}0&{}0&{}0\\ -1&{}1&{}0&{}0&{}0&{}0\\ 0&{}0&{}0&{}0&{}0&{}0\\ 0&{}0&{}0&{}0&{}0&{}0\\ 0&{}0&{}0&{}0&{}0&{}0\\ 0&{}0&{}0&{}0&{}0&{}0\\ \end{array} \right) \end{aligned}$$

for all six distances among the four corners of the *same* square configuration. (In this matrix some of the 0’s are exact and some are only approximate, corresponding to distances that vary relatively less and less as the variability in the direction of the factor indicated by the arrows drops lower and lower.) On the left, the two distances that have equal variances and a perfect correlation of $-1$ are the two diagonals of the square; on the right, they are a pair of parallel edges. The difference between this pair of exemplary models on four landmarks does not manipulate the mean locations of the landmarks, only their numbering. (The essence of the contrast is that the segments whose lengths bear the negative correlation intersect in the first instance but are disjoint in the second.) In spite of arising from the same mean form *and having the same covariance structure among the set of all six relative distances,* the shape phenomena in question are completely unrelated as biological patterns. On the left, we see a transformation that would be reported by thin-plate spline as a uniform change; on the right, no uniform term, but instead a pure growth-gradient (linear dependence of the affine derivative along some transect of the form). Clearly the locations of the average landmarks and even the numbering of those locations matter for interpretation of these covariances in terms of biology, but that information is not accessible to the factor analysis machinery or the associated permutation tests by which current approaches customarily deal with it. In other words, the covariances of the distances *per se* are not sufficient to make any sense of variations in these configurations—the mean locations must somehow be brought into the analysis.

A further caveat applies with even greater force to *any* pattern by which the six distances on landmark pairs of a starting square are claimed to change. If the landmarks are to lie in a plane at all, the distances must satisfy a complicated polynomial condition that seems intuitively inaccessible: the condition

1 $$\begin{aligned} \left| \begin{array}{ccccc} 0&{}1&{}1&{}1&{}1\\ 1&{}0&{}d_{12}^2&{}d_{13}^2&{}d_{14}^2\\ 1&{}d_{12}^2&{}0&{}d_{23}^2&{}d_{24}^2\\ 1&{}d_{13}^2&{}d_{23}^2&{}0&{}d_{34}^2\\ 1&{}d_{14}^2&{}d_{24}^2&{}d_{34}^2&{}0\\ \end{array} \right| = 0, \end{aligned}$$

where $d_{ij}$ is the measured distance between landmark $i$ and landmark $j$. If there are more than four landmarks, this must be true for every subset of four. The determinant is actually 288 times the squared volume of the tetrahedron on the six edges. In this form it is called the *Cayley-Menger formula* for that squared volume. (But the formula is remarkably old—it originated with Piero della Francesca, the fifteenth-century Italian geometer and painter, although in a different notation, the determinant $\vert \cdot \vert$ not having been invented yet.) Any representation of “all the distances” among four or more landmarks in two dimensions, or five or more in three dimensions, necessarily lies on a curving subsurface of the corresponding multivariate space, and hence *cannot* be described by a multivariate Gaussian, certainly not one of full rank.

**Fig. 8:**
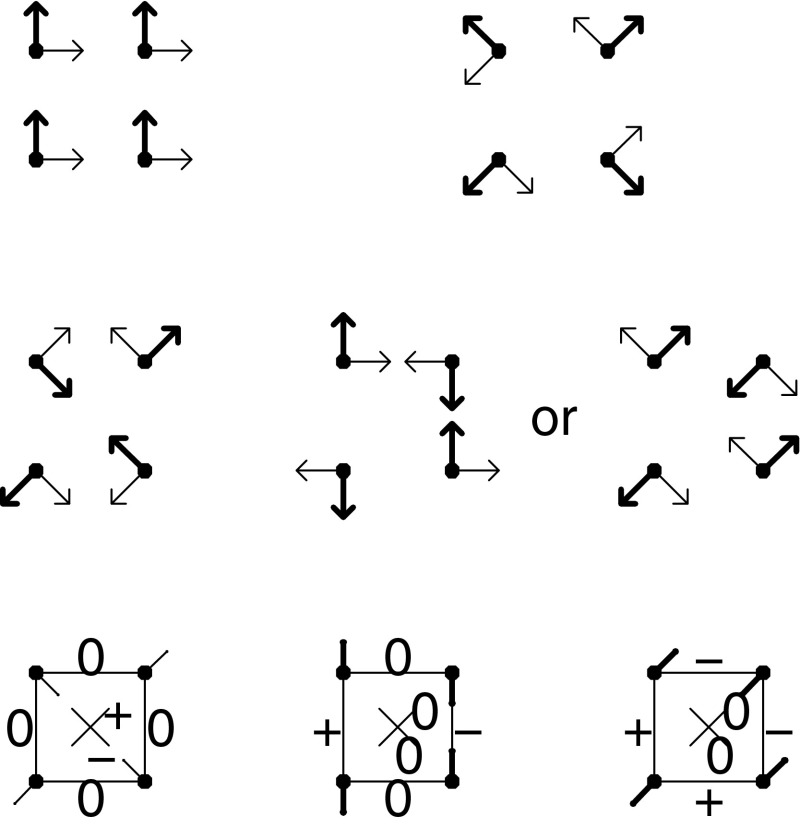
Dimensions of the shape space for variations around an exact square. (*top*) The four patterns of coordinated change in the Procrustes coordinates of landmarks around a square starting form that must have zero variance. They are drawn two per panel, *thin arrows* or *thick arrows*, at $90^\circ$ both in the full Procrustes space and at each landmark separately. (*middle*) There remain four dimensions which can be notated using the little vectors here. The second set, arising from the first principal warp of the bending-energy matrix for this square, is drawn to two different bases focusing on different patterns of changes in pairwise landmark distance, but the two-dimensional subspace they span (the purely nonaffine transformations) is the same. (*bottom*) Differentials of the six edge-lengths for three of the patterns in the middle row (those drawn with the *solid arrows*). $+$: distances that increase with increase in the component drawn. $-$: distances that decrease. $0$: distances that do not change to first order in the change of the component score. The *left* and *center* panels of this row are the same simulations already shown at the *bottom* of Fig. 7

Back in two dimensions of landmark coordinates, the meaningful dimensions of shape changes in Procrustes space for a square mean form necessarily zero out the four patterns of joint coordinate variation shown in the top row of Fig. 8, while leaving the other four dimensions, those shown in the middle row, free to vary. In the bottom row I have interpreted three of these middle patterns (the three drawn in solid arrows) in terms of the effective differential for each edge of the original square—increase, decrease, or invariance (to first order, anyway). There are six such patterns in total, but only four dimensions, so the patterns must be correlated across the modes. This means that we cannot diagnose the kind of transformation we are looking at just by examining the signs of changes of edges. We have to know where the landmarks are, too.

Such paradoxes and counterexamples can be extended *ad libitum.* We make little progress toward the understanding of patterns of shape change by examining covariance structures alone. If integration is to be studied from a biologically fruitful point of view, it must be based in some formal combination of the information in the mean form and the information in the shape covariances. That combination is precisely what the algorithms in the next section produce.

## A Theorem with Its Corollary Algorithms

This section sketches the mathematical basis for the formal statistical-geometrical study of integration and its associated data-analytic algorithms. The methodology turns out to spring from the self-similarity property already noted in connection with Figs. 1, 2, 3, 4 and 5. This invariance—the reason that bending-deflated versions of Procrustes distributions produce self-similar features within the corresponding nonaffine subspaces—will drive a general algorithm for parameterizing real-world data sets in terms of their scaling properties. The algorithms involved in producing these distributions and the relative intrinsic warps that summarize them are set out in detail. “Integration” will be a biological interpretation of the rejection of self-similarity when the regression slope produced by Algorithm III below is greater than 1 in absolute value. In Sect. "Visualizing Integration: Three Examples" the technology will be extended to include displays that demonstrate the range of scales within which some real data sets prove to be self-scaling or, when they are not, the representation by polynomial growth-gradients of the features by which they differ from that model.^2^

Let us briefly review the standard notation for thin-plate splines and their descriptors as first published by Bookstein (1989). In this notation, let $U$ be the function $U(r)=r^2\log r$, and write $P_i=(x_i,y_i),i=1,\ldots ,k$, for $k$ points in the plane. With $U_{ij}=U(P_i-P_j)$, build up matrices

2 $$\begin{aligned} K=\left( \begin{array}{cccc} 0&{}U_{12}&{}\ldots &{}U_{1k}\\ U_{21}&{}0&{}\ldots &{}U_{2k}\\ \vdots &{}\vdots &{}\ddots &{}\vdots \\ U_{k1}&{}U_{k2}&{}\ldots &{}0\\ \end{array}\right) ,\quad Q=\left( \begin{array}{ccc} 1&{}x_1&{}y_1\\ 1&{}x_2&{}y_2\\ \vdots &{}\vdots &{}\vdots \\ 1&{}x_k&{}y_k\\ \end{array} \right) , \end{aligned}$$

and

3 $$\begin{aligned} L= \left( \begin{array}{cc} K&{}Q\\ Q^t&{}O\\ \end{array}\right) ,\quad (k+3)\times (k+3), \end{aligned}$$

where $$O$$ is a $$3\times 3$$ matrix of zeros. Write $$H=\bigl (h_1\ldots h_k\,0\,0\,0\bigr )^t$$ and set $$ V = \bigl (v_1\ldots v_k\,a_0\,a_x\,a_y\bigr )^t= L^{-1}H$$. Then the thin-plate spline $$f(P)$$ having heights (values) $$h_i$$ at points $$P_i=(x_i,y_i),$$$$i=1,\ldots ,k$$, is the function

4 $$\begin{aligned} f(P)=\sum _{i=1}^{k}v_iU(P-P_i)+a_0+a_xx+a_yy. \end{aligned}$$

This function $f(P)$ has three crucial properties:

1. $f(P_i)=h_i$, all $i$: $f$ interpolates the heights $h_i$ at the landmarks $P_i$.
2. The function $f$ has minimum *bending energy* of all functions that interpolate the heights $h_i$ in that way: the minimum of
  5 $$\begin{aligned} \int \!\!\!\int _{\mathbf{R}^2} \left( \left( {\partial ^2f\over \partial x^2}\right) ^2 +2\left( {\partial ^2f\over \partial x\partial y}\right) ^2 +\left( {\partial ^2f\over \partial y^2}\right) ^2\right) \end{aligned}$$
  where the integral is taken over the entire picture plane. (The word “bending” is borrowed from continuum mechanics, where a corresponding expression describes the *actual* bending energy of an idealized thin metal plate originally flat but now clamped at locations corresponding to the heights over the landmarks.)
3. The value of this bending energy is
  6 $$\begin{aligned} {1\over 8\pi }V^tKV={1\over 8\pi }V^t\cdot H ={1\over 8\pi }H_k^tL_k^{-1}H_k, \end{aligned}$$
  where $L_k^{-1}$, the *bending energy matrix,* is the $k\times k$ upper left submatrix of $L^{-1}$, of rank $k-3,$ and $H_k$ is the initial $k$-vector of $H$, the vector of $k$ heights.

The bending energy matrix’s three eigenvalues of zero correspond to height surfaces that are exact mathematical planes: the height surfaces $f: (x,y)\rightarrow a_{0}+a_{1}x+a_{2}y$. Eigenvectors for the other $k-3$ eigenvalues have diagrams that look bent. These nonzero eigenvalues are conventionally sorted in increasing order, from least to greatest eigenvalue. Whenever eigenvalues are distinct the corresponding eigenvectors are orthogonal with respect to the sum of squared displacements $h$ (equivalently, with respect to squared Procrustes length). Each eigenvalue is the “specific bending” of its eigenvector, meaning, $8\pi$ times the actual bending energy of the interpolant as extrapolated to unit Procrustes length.

In the application to two-dimensional landmark data, we compute two of these splined surfaces, one ($$f_x$$) in which the vector $$H$$ of heights is loaded with the $$x$$-coordinate of the landmarks in a second form, another ($$f_y$$) for the $$y$$-coordinate. Then the first of these spline functions supplies the interpolated $$x$$-coordinate of the map we seek, and the second the interpolated $$y$$-coordinate. It is easy to show (Bookstein 1989) that we get the same map regardless of how we place the ($$x,y)$$ coordinate axes on the picture. For any such coordinate system, the resulting map $$(f_x(P),f_y(P))$$ is now a deformation of one picture plane onto the other which maps landmarks onto their homologues and has the minimum bending energy of any such interpolant. The bending energy of a grid is now the scalar sum of the bending energies in the $$x$$-coordinates and $$y$$-coordinates of the target configuration separately. To the trained eye, the grid looks “as smooth as it can be” given where the landmarks have to go—it *looks* like it is minimizing some sort of net bending, which is just what it is actually doing. The *affine* or *uniform transformations* are the formulas $$(x,y) \rightarrow (a_{0x}+a_{1x}x+a_{2x}y, a_{0y}+a_{1y}x+a_{2y}y)$$. Maps of this class continue to have bending energy zero.

The basic mathematical result on which I am relying is a *theorem* brought to our attention by Kent and Mardia in an underappreciated paper of 1994 showing how the thin-plate spline of geometric morphometrics, a graphical style still somewhat unfamiliar at the time, serves also as the solution of a certain problem of *kriging,* which is actually a technique for the optimal prediction of spatial random fields.^3^ A random field $Y(t)$ in $d$ Cartesian dimensions is called *self-similar* for some degree $-\alpha$ if for any positive $s$, which will be identified below with the scale of a biometrical feature, the distribution of $s^\alpha Y(st)$ is the same as that of $Y(t).$ (I have omitted some niceties of notation.) The thin-plate spline in two dimensions turns out to satisfy this equation with $\alpha =-1.$ In the sequel we will limit our attention to the “intrinsic random fields,” those considered without reference to the linear (affine) term. This constraint is identical in its logic to the approach in the temporal domain that studies Brownian motion without paying any attention to its starting location, since, technically speaking, the mean of a Brownian motion is simply irrelevant, and when followed over increasingly long time intervals its variance becomes greater and the retrospective estimate of the starting value steadily more and more imprecise.

If the thin-plate spline is considered as an example of a prediction function, the covariance between values observed and values predicted is closely related to the entries $\sigma (r)= r^2\log ~r$ of the matrix $K$ in Eq. (2). As noted on page 65 of Mardia et al. (2006), this covariance function satisfies the identity $\sigma (sr)\equiv s^2 \sigma (r)$ up to an even quadratic polynomial. (We have, in fact, $(sr)^2\log\,(sr) = s^2 (r^2 (\log r+\log s)) = s^2 (r^2\log r)+ r^2(s^2\log s),$ which differs from $s^2 \sigma (r)$ only by a scalar multiple of $r^2.$) Hence, within the subspace of trend-free regression splines, $\sigma (r)$ and $\sigma (sr)$ yield the same predictions. Another way to state this is that the whole thin-plate spline approach is invariant under arbitrary isotropic changes of Cartesian coordinate system (translations, rotations, rescaling). It was already obvious (see Bookstein 1989) for translations and rotations; the equivalence of $\sigma (r)$ and $\sigma (sr)$ constitutes the same verification in respect of scaling.

The theorem at which Kent and Mardia (1994) arrive is that the thin-plate spline is a solution of the kriging problem, meaning that it is an optimal predictor in a sense different from that of Eq. (5). There, the spline was treated as a function of the position being predicted, with the data $h$ fixed. In kriging, the same formula is treated as a linear combination of variable data $h$, with the prediction target fixed. The concept of self-similarity arises in the kriging context, most commonly in geostatistics, where it relates prediction errors at different sites. It is the spectrum of the bending-energy matrix that permits this concept to transfer to the domain of interpolation maps (deformation grids, D’Arcy Thompson’s “Cartesian transformations”), which is where today’s biologist usually encounters them.

This equivalence of splined grids and kriging-based prediction can be reworded in a more biologically accessible language. Our intuition tells us that, qualitatively speaking, nearby pairs of landmarks should be expected to covary in position more strongly than landmarks at greater distance. Such a statement is not yet ready for prime time, as we didn’t specify how position was to be quantified. Rephrase, then: *in a coordinate system in which one of the landmarks is fixed*, we expect that the position of the second landmark with respect to the first landmark has a variance that is, in general, smaller as its distance from the fixed landmark shrinks. But we still aren’t thinking with sufficient precision to satisfy the geometer. For that notion of “position” to make sense, there has to be an orientation assigned to that coordinate system, not just a center. So actually we needed to be talking about three landmarks, not two. And yet there is *still* something unsatisfying about this way of thinking, because if the reference direction for the coordinate system we are imagining is set at some finite distance (e.g., the other end of the long axis of the form), it may have rotated (perhaps by quite a large angle) away from the orientation most relevant to the local comparison we are trying to quantify. Sorting out all of these caveats, it appears that we need *four* local landmarks or semilandmarks, not three: two to set a reference scale and direction, and two others to be assessed for variability of both that scale and that direction. The appropriate geometric reference structure, then, is a square in one specimen, and something not quite a square in another specimen; and our quantification is the extent to which the two parallel edges that are the same vector in the one specimen are the same vector in the other specimen. It is *this* variation—the variation of the location of the fourth vertex of a small quadrilateral given the prediction from the locations of its other three vertices—that we expect to grow smaller as the starting square grows smaller.^4^ In the world of deflated isotropic Procrustes distributions, this discrepancy grows smaller with a variance that is precisely proportional to the area of the square. This is the model of self-similarity that this paper will rely on as a null model separating the relatively more integrated data sets from those that are relatively more disintegrated.

With this machinery in place it is now possible to set out the algorithms for all the figures here. Write $$B$$ for the bending-energy matrix $$L_k^{-1}$$ of Eq. (6) as computed at the Procrustes average shape, $$E$$ for the vector of nonzero eigenvalues of $$B,$$ and $$W$$ for the corresponding eigenvectors (the partial *W*arps) in matrix form. The columns of $$W$$ should be normalized to geometric length 1, so that $$B=W\,\mathrm{diag}(E)W^t.$$ Also, write $$\mu = (P_1,\ldots, P_k)$$ for the list of landmark locations of the mean shape (Fig. 3, upper left), $$Cdist$$ for the isotropic Gaussian distribution $$N\bigl (\mu ,\sigma ^2I_{2k}\bigr )$$ around $$\mu $$ in digitizing space, $$Ddist$$ for the analogous distribution based on observed landmark locations from some data set, and $$Pdist$$ (with mean $$Pmean$$) for the matrix of shape coordinates arising from Gower’s generalized procrustes analysis (GPA) as applied to the samples $$Cdist$$ or $$Ddist$$, whichever drives the computation at hand (Fig. 3, upper right). Standardize these Procrustes means $$\mu $$ as follows: when they are vectorized as lists of $$2k$$ Cartesian coordinates $$(x_1,y_1,x_2,y_2,\ldots ,x_k,y_k)$$, we require $$\sum x_i = \sum y_i = \sum x_iy_i = 0,$$$$ \sum (x_i^2+y_i^2) = 1$$ (meaning: $$\mu $$ is centered, its Centroid Size is 1, and it has been rotated to principal axes horizontal and vertical). Write $$\alpha = \sum x_i^2$$ and $$\gamma = 1-\alpha =\sum y_i^2$$ — the central moments of the mean configuration in its principal directions. (This is a different $$\alpha $$ from the $$\alpha $$ in the theory of self-similarity; I use the symbol here for consistency with the earlier literature.)

Let $nonaff$ be the operation that projects the uniform term out of distributions like $Cdist$ or $Ddist$: the operation that partials out the projections corresponding to the two linear combinations

7 $$\begin{aligned} \left( \begin{array}{ccccccc} \alpha y_1 &{} \gamma x_1 &{} \alpha y_2 &{} \gamma x_2 &{} \ldots &{} \alpha y_k &{} \gamma x_k \\ -\gamma x_1 &{} \alpha y_1 &{} -\gamma y_2 &{} \alpha y_2 &{} \ldots &{} -\gamma x_k &{} \alpha y_k\\ \end{array}\right) . \end{aligned}$$

(These are the terms uniform.x and uniform.y drawn as deformations of the mean polygon in the second row of Fig. 1 of the Prologue.)

Then the fundamental computations needed for all of the data-based diagrams here, whether from empirical data or from simulations, are as follows.

I. *Partial warp scores.* For each case $j$ between 1 and $n$ and each partial warp index $l$ between 1 and $k-3,$ this is the quantity $(W_l\cdot Pdist_j)$ where the operator $\vert \cdot \vert$ is taken in the ordinary sense of a dot product of a vector of real numbers (the elements of $W_l$) by a vector of complex numbers (the locations of the shape coordinates of the $j$th specimen in $Pdist$). The dot product can be taken with respect to the original distribution $Pdist$ instead of the nonaffine part $nonaff(Pdist)$ because the partial warps are orthogonal to the uniform terms of Eq. (7).
II. *Deflation.* For any shape distribution $Pdist$ on $k$ landmarks for $n$ specimens, the bending-energy matrix at the Procrustes mean has $k-3$ nonzero eigenvalues $E$ with eigenvectors $W,$ a matrix $k\times (k-3).$ The *deflation* of the distribution $Pdist$ consists of replacing the observed Procrustes shape distribution $Pdist$ with the distribution $defl$ where, case by case,
  8 $$\begin{aligned} defl=Pmean+\sum _{l=1}^{k-3} \sqrt{{E_1\over E_l}} (W_l\cdot Pdist)W_l. \end{aligned}$$
  Here the quantities $(W_l\cdot Pdist)W_l$ are the partial warp scores of Algorithm I multiplied by the corresponding columns of the matrix $Pdist,$ and the prefactor is the scaling by the inverse square root of specific bending energy (with respect to the partial warp of largest scale, $l=1$ in Eq. (8), which is evidently left unchanged). Like the Procrustes mean shape $\mu$, each entity of the distribution $defl$ is conventionally vectorized as $2k$ real numbers, but the sum in Eq. (8) is over only $k-3$ expressions, not $2k-6$, because the notation is treating the $Pdist$ terms as complex numbers. This is the distribution exemplified in Fig. 3 (lower left). By construction the uniform component of $defl$ must be zero, as it is zero for each of the partial warps separately. A similar convention will apply to the modified equation (8′) below.
III. *Parameterization.* Plots like those in Fig. 12 are log-log regressions of the variances of the $k-3$ nonaffine partial warp scores $(W_l\cdot Pdist)$ on the bending energies $E_l$ warp by warp. The slope of such a regression is compared to the fixed value of $-1$ to assess whether the data structure is as expected on the hypothesis of a self-similar random field or is more integrated or more disintegrated than that. I often restrict these regressions to subsets of the relative warps thresholded for some subrange of the larger spatial scales. For the textbook Procrustes shape distribution $Cdist,$ the “isotropic offset Gaussian distribution,” this slope is expected to be zero: to a spherical distribution in shape space corresponds an expectation of equal variances on any suite of orthogonal components spanning that space, regardless of their relation to the mean form. It is even possible for the slope to be positive, the same way that errors in an Ornstein-Uhlenbeck temporal process are anti-autocorrelated; that would correspond to the art of caricature. A slope of $-1$ for partial warp variance against bending energy embodies the model of self-similarity demonstrated in the Prologue that separates our two regimes of biologically contrasting organization, the integrated and the disintegrated.
IV. *Relative intrinsic warps.* The *relative intrinsic warps* (RIW’s) are the principal components of the distribution $defl.$ This means: compute the $2k-6$ nontrivial principal components of the covariance matrix of the deflated shape coordinate $2k$-vectors $defl,$ expressed in the basis of the deflated partial warps $W_l\sqrt{E_1/E_l}$ as in Eq. (8). The technical name for such a procedure is a *relative eigenanalysis* (Bookstein and Mitteroecker 2014). These are the patterns of an integrated deformation that emerge as a hierarchical list, orthogonal with respect to bending energy, of the features manifesting more bending than expected given their specific bending energy, which is to say, their geometric scale. If the RIW’s are drawn using the undeflated warps $W_l$ instead of the deflated warps $W_l\sqrt{E_1/E_l}$, any modules accompanying the integrated analysis will be shown with less attenuation. In the language of a neighboring field (medical image analysis), the relative eigenanalysis is serving as a smoothly tapered low-pass filter for the representation of spatial patterns of deformation, on the assumption that a regression slope below $-1$ at step III justifies a focus on the spatial trends of largest scale (within the nonuniform subspace).

The RIW’s here were already introduced in Section 7.5 of Bookstein (1991) without being restricted to the integrated transformations. There they were called “relative warps,” with the deflation step left unmentioned, as if obvious. However, that version was shortly superseded by a computation omitting the deflation step. The simpler computation was introduced by Kent (1994) and shortly thereafter became more widely disseminated as a result of its exposure in Dryden and Mardia (1998) and its encoding in Paul O’Higgins’s morphologika statistical package for physical anthropologists. My original version, the one now called “relative intrinsic warps,” was acknowledged in a footnote in Rohlf (1993) as the case of “relative warps with $\alpha =1.$” As far as I know, the present paper is its first journal appearance anywhere. Part of the problem might be that the original publication emphasized the grid interpretation of the dominant warps one by one rather than examining the whole sequence of their eigenvalues as in the presentation here. Another reason for the burial of the original suggestion was the unfortunate decision to concentrate on the estimation of the integrated pattern of RIW1 per se instead of on the estimation of $\alpha ,$ which I show here to be the more fundamental parameter and which, as a scalar, is relatively easier to triage and discuss.

In passing, note how the deflation protocol of Algorithm II helps buffer the principal-components computations of Algorithm IV against what would otherwise be a standard paradox of principal components analysis. Whether the variables being analyzed are ordinary size measures or instead shape coordinates, standard principal components are altered when some variables are duplicated or nearly duplicated. For measured lengths, this could be as simple as including intentionally redundant sets of distances, such as the height of the head computed as the distance from the vertex to each of the wide range of possible “horizontal baselines” offered in Martin (1928), Figure 295. For shape coordinates, it would be the analogous effect of landmarks that are much more densely sampled in some parts of the anatomy than in others. The deflation approach, in contrast, is strikingly less sensitive to these differences of density. Closely spaced sublists of landmarks are represented by a single dimension for their shared information content (in effect, their own average location) together with additional partial warps at much greater bending energy corresponding to the displacements between these near neighbors. Those additional dimensions will be deflated to nearly zero weight by Algorithm II. As there is in fact no rigorous protocol according to which landmarks are to be distributed over an anatomy (a problem that is even worse for data that are represented by semilandmarks along curves or surfaces, for which arbitrariness of spacing is part of the actual definition), it’s good news that the proposed replacement for relative warps hardly shares at all the dependence of the Kent method on arbitrary decisions about spacing. The situation would be the same if, when a new length measure is being considered for a factor study, it appears in the analysis in the form of its unique variance, its value after the regression on all of the other measures already in the analysis; but this is not how principal component analysis actually works. (It is, however, the version of factor analysis named *image analysis* developed by Guttman 1953.)

### *In three dimensions.*

For three-dimensional data (Cartesian coordinate triples), the kernel function $U(r)$ is now $\vert r\vert ,$ ordinary Euclidean distance, and otherwise formulas (2)–(6) for the thin-plate spline are essentially the same except for changes of subscripting. Explicitly, one has

2′ $$\begin{aligned} K= \left( \begin{array}{cccc} 0&{}\vert P_1-P_2\vert &{}\ldots &{}\vert P_1-P_k\vert \\ \vert P_2-P_1\vert &{}0&{}\ldots &{}\vert P_2-P_k\vert \\ \vdots &{}\vdots &{}\ddots &{}\vdots \\ \vert P_k-P_1\vert &{}\vert P_k-P_2\vert &{}\ldots &{}0\\ \end{array}\right) ,\quad Q= \left( \begin{array}{cccc} 1&{}x_1&{}y_1&{}z_1\\ 1&{}x_2&{}y_2&{}z_2\\ \vdots &{}\vdots &{}\vdots &{}\vdots \\ 1&{}x_k&{}y_k&{}z_k\\ \end{array}\right) , \end{aligned}$$

and

3′ $$\begin{aligned} L= \left( \begin{array}{cc} K&{}Q\\ Q^t&{}O\\ \end{array}\right),\quad (k+4)\times (k+4), \end{aligned}$$

where $$O$$ is now a $$4\times 4$$ matrix of zeros. $$H$$ is now $$\bigl (h_1\ldots h_k\,0\,0\,0\,0\bigr )^t$$ and if we write out $$L^{-1}H$$ as a subdivided vector $$\bigl (v_1\ldots v_k\,a_0\,a_x\,a_y\,a_z\bigr )^t,$$ the thin-plate spline $$f(P)$$ taking on values $$h_i$$ at points $$P_i=(x_i,y_i,z_i),$$$$i=1,\ldots ,k$$, is now the function

4′ $$\begin{aligned} f(P)=\sum _{i=1}^{k}v_iU(P-P_i)+a_0+a_xx+a_yy+a_zz. \end{aligned}$$

This function $f(P)$ continues to have the same crucial properties as its two-dimensional analogue. $f(P_i)=h_i$ for each landmark $P_i$. Over all the functions that interpolate the heights $h_i$ in that way, $f$ has the minimum of bending energy, now the triple integral

5′ $$\begin{aligned} \int \!\!\!\int \!\!\!\int _{ \mathbf{R}^3} \left( \left( {\partial ^2f\over \partial x^2}\right) ^2 +2\left( {\partial ^2f\over \partial x\partial y}\right) ^2 +\left( {\partial ^2f\over \partial y^2}\right) ^2 +2\left( {\partial ^2f\over \partial x\partial z}\right) ^2 +2\left( {\partial ^2f\over \partial y\partial z}\right) ^2 +\left( {\partial ^2f\over \partial z^2}\right) ^2 \right) \end{aligned}$$

taken over all space. And the value of this bending energy remains a multiple of the quadratic form

6′ $$\begin{aligned} H_k^tL_k^{-1}H_k, \end{aligned}$$

although the matrix $L_k^{-1}$ is now negative semidefinite rather than positive semidefinite.

Corresponding to this interpolant is the self-similar deflation of the three-dimensional Procrustes shape coordinates according to the spectrum of $L_k^{-1}$ where, owing to the change in the kernel function $U$ to the linear term, the value of $-\alpha$ in the self-scaling equation is $-{1\over 2}$ instead of $-1,$ and the deflation that ensues must be by the ratio of energies ${{E_1}\over {E_i}}$ rather than its square root. Explicitly, for three-dimensional data, one has

8′ $$\begin{aligned} defl=Pmean+\sum _{l=1}^{k-4} {E_1\over E_l} (W_l\cdot Pdist)W_l. \end{aligned}$$

where each multiplicand $W_l$ is now a triplex of $k$-vectors for the $l$th eigenvector of $L_k^{-1}$ in the $x$-, $y$-, and $z$- slots, respectively, of the full Procrustes shape coordinate vector, and where the square-root symbol of Eq. (8) has been deleted. Just as Eq. (8) resulted in a nonaffine space of rank $2k-6$ for two-dimensional data, corresponding to the annihilation of two dimensions of uniform shape change, so Eq. (8′), the variant for three-dimensional data, results in a nonaffine space of dimension $3k-12$, versus the rank of $3k-7$ for the Procrustes shape coordinates in full. But there is no convenient equivalent of the useful pair of formulas in Eq. (7) for the five dimensions of uniform transformations in the context of three-dimensional data, and the interpretation of the RIW’s as relative eigenvectors must be altered a bit (the reference matrix now being the square of the bending energy matrix, not the bending energy itself).

**Fig. 9:**
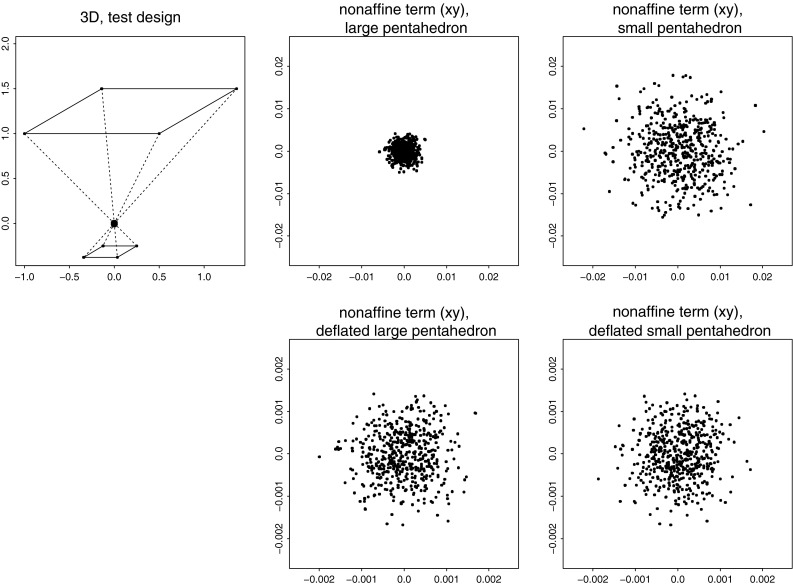
A simple example of the effect of deflation for three-dimensional data. (*upper left*) The test design, consisting of two square pyramids of different scales to the same apex. (*upper center and right*) In an isotropic Mardia–Dryden distribution of Procrustes shapes for this mean form, the amplitude of the nonuniform component for the smaller pentahedron is four times that for the larger one. (*lower center and right*) After deflation by Eq. (8′) as per the text, the two pentahedra show nonaffine variation of the same amplitude in spite of the fourfold difference of their geometric scales

Figure 9 shows an example of the three-dimensional deflation protocol corresponding in its simplicity to the scheme of Fig. 1. The “test design,” upper left panel, is just a pair of pentahedra of the same shape (inspired by the pyramids at Giza), the larger one exactly four times the scale of the smaller. Because transformations of tetrahedra can always be modeled as uniform, the “nonaffine component” of a set of pentahedral shapes can be parameterized as a three-vector. In the usual (offset isotropic) Procrustes simulations this particular three-vector is spherical for small shape variations, so we can graph any orthogonal pair of its dimensions. In this example, samples of 500 have been drawn from the corresponding spherical Gaussians of low standard deviation in the covering $\mathbf{R}^{27}$. The remaining two figures of the top row show the $x$- and $y$- coordinates of this nonaffine component for the two pentahedra of the design after the transformation to Procrustes shape coordinates. These two dimensions are arbitrary except that, insofar as they are orthogonal, they confirm the sphericity that follows from the symmetries of the simulation. Before deflation, the variance for the smaller pyramid (upper right) is four times the variance for the larger one (upper center), corresponding to the inverse of the fourfold ratio of their scales.

The test configuration of the two pentahedra has five nontrivial eigenvalues of bending energy, corresponding to four dimensions of deflation by ratios to the least negative of these. Following Eq. (8′), each of these ratios is applied three times, once to the $x$-subscripted shape coordinates along the direction of the eigenvector, once to the $y$’s, and once to the $z$’s. After deflation, we repeat the extraction of the two identically shaped pentahedra and the construction of the nonaffine component of shape variation for each. These three-vectors are still spherically distributed, and now typical sectional scatterplots show the same variance (lower row) in spite of the factor of four in scale of the mean configuration. In other words, deflation works just as well for three-dimensional data as for two-dimensional data, as long as one deletes the square-root operation in formula (8). It is a provocative thought that the difference between analysis in two dimensions and analysis in three might reduce to this single editorial alteration, the elimination of the radical. It accommodates the fundamental change in the meaning of the manifold of directions around a point between the two settings. For two-dimensional data, this set of directions is a circle; in three dimensions, it is a spherical surface instead.

## Visualizing Integration: Three Examples

This section reanalyzes three extant data sets from the point of view of the preceding concerns. One of the data sets shows strong integration, one seems indistinguishable from the featureless state of self-similarity, and one hints at the possibility of disintegration within our species but self-similarity across our clade. The landmark schemes and data sets involved here have been published before, and all three were reviewed in some detail in Bookstein (2014), though not in the light of this concern for self-similarity.

### *Example 1*

Vilmann’s rodent growth data

**Fig. 10:**
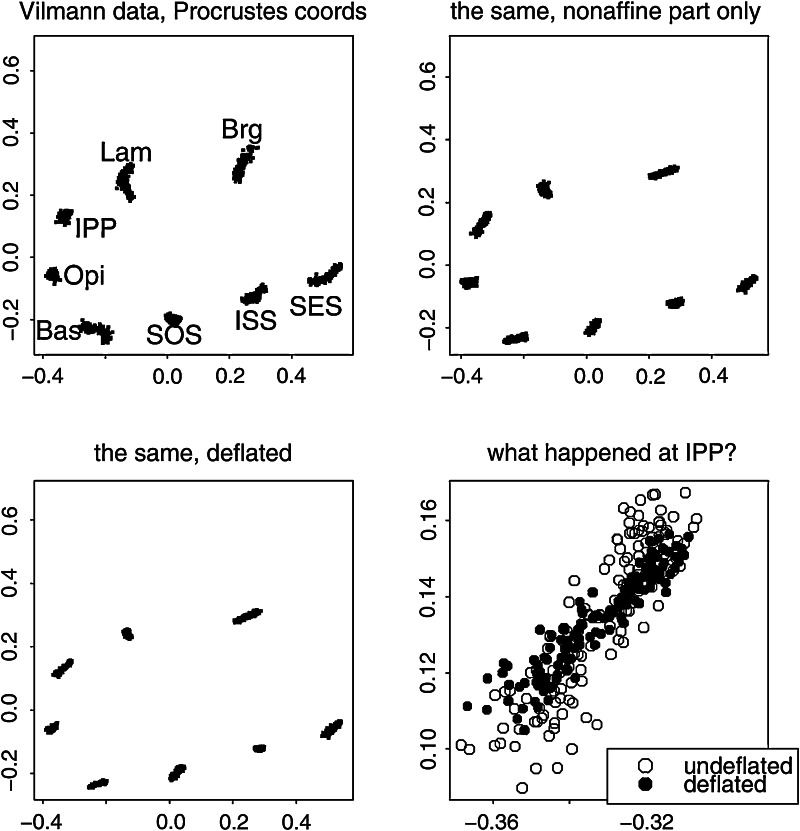
Three shape coordinate scatters for the Vilmann rodent skull octagons. (*upper left*) Ordinary Procrustes shape coordinates. (*upper right*) Without the uniform component (consistent height reduction plus a temporally inconsistent shearing). (*lower left*) Deflated coordinates from Algorithm II. Landmarks: *Bas* Basion, *Opi* opisthion, *IPP* intraparietal point, *Lam* Lambda, *Brg* Bregma, *SES* Sphenoethmoid synchondrosis, *ISS* intersphenoidal synchondrosis, *SOS* spheno-occipital synchondrosis. The variation in apparent amplitudes of the landmark-by-landmark plots is close enough to distance from the centroid to be captured by the quadratic analysis in Figs. 14 and 15

**Fig. 11:**
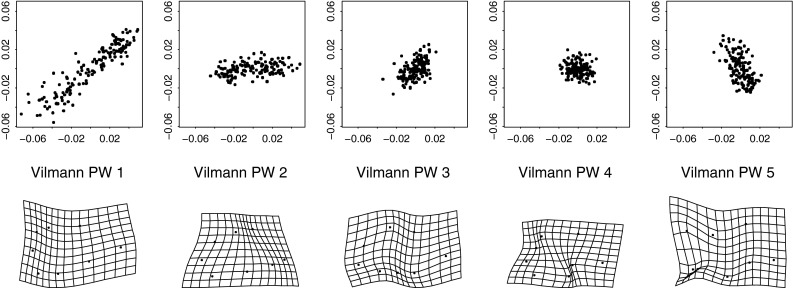
The results of Algorithm I show a steady drop of variance with partial warp score for all except the last (most highly bent, smallest scale) of these. This strongly suggests the possibility of an integrated growth gradient accompanied by a local shape feature

**Fig. 12:**
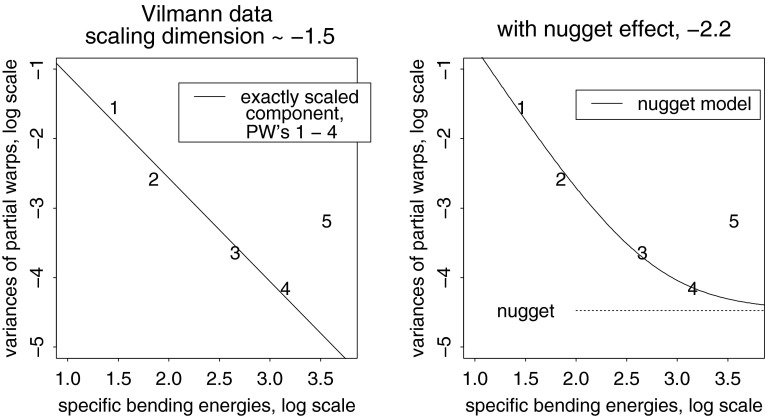
Two estimates of the scaling dimension of rodent skull growth. (*left*) Regression of log partial warp variance on log bending energy across all five dimensions. (*right*) Regression for the first four partial warps only, plus a nugget term for digitizing error, results in a scaling of $-2.2,$ satisfactorily different from $-1.$ See text

**Fig. 13:**
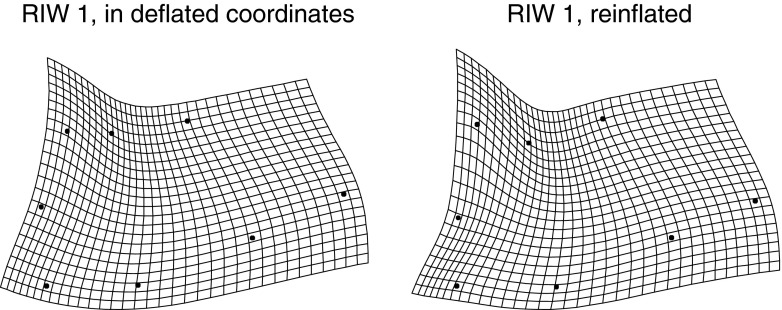
The single interpretable relative intrinsic warp for these rodent data. (*left*) In the deflated coordinate system of Fig. 10, *lower left*. (*right*) “Reinflated” back to Procrustes units. The impression of two features, one a large-scale integration and one local to the IPP, leaps to the eye in the reinflated version

**Fig. 14:**
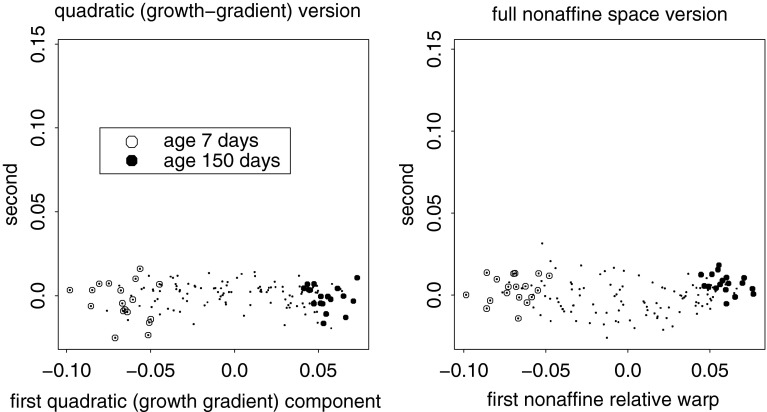
Closing in on the large-scale integrated component for the Vilmann data. (*left*) Only one principal component rises above spherical noise for the six dimensions of quadratic trend. This would be strong evidence of integration regardless of the more sophisticated regression evidence in Fig. 12. (*right*) For the entire 10-dimensional nonaffine shape subspace, the first relative warp is indistinguishable from the quadratic (growth-gradient) version at left ($r\sim 0.999$)

**Fig. 15:**
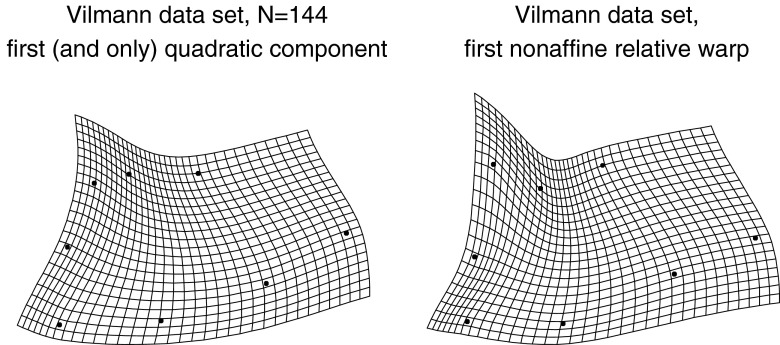
The large-scale quadratic (integrated) trend is indistinguishable from the deflated relative intrinsic warp in Fig. 13, while the first principal component of the nonaffine shape coordinates is indistinguishable from the combination of this component with a local effect at IPP, the same pattern as the reinflated first RIW from Fig. 13. The grid on the left has the same second derivative at every point, and hence could be considered as integrated as any uniform transformation (for which it is the *first* derivative that is similarly unchanging)

The likeliest place to find integration would be a region characterized, in Melvin Moss’s felicitous phrase, as a “functional matrix,” a coherent anatomical domain balancing diverse functional criteria that persist over a growth trajectory. One such data set is the octagon of landmarks circumscribing the developing brain in the midplanes of 21 rodents (of which the data from 18 are used here) that were radiographed cephalometrically at ages 7, 14, 21, 30, 40, 60, 90, and 150 days after birth by the Danish morphologist Henning Vilmann; the landmarks were digitized by Moss himself. These data were first used to illustrate morphometric techniques in Bookstein (1984) and were listed *in extenso* as an Appendix to Bookstein (1991). For a diagram of this landmark scheme, eight points on 21 growing rodent skulls at eight ages, see Bookstein (2014), Figure 6.8. Analysis by the principles of this paper is the concern of Figs. 10, 11, 12, 13, 14 and 15 here. For a different approach to this same data set, centered on the within-age covariances instead of the growth trajectories, see Bookstein and Mitteroecker (2014).

At upper left in Fig. 10 we see the conventional Procrustes shape coordinate plot of these octagons. There is a substantial uniform component to the growth trajectory, visible most clearly at the landmark Lambda: the combination of a consistent drop in overall height of the calva relative to the cranial base length with a shearing along this axis that reverses from the age interval 7–30 days to the age interval 30–150 days. After this uniform component is removed, for the reasons given in Sect. "A Theorem with Its Corollary Algorithms", we see a striking pattern involving relative changes of length along the upper and lower borders of this octagon, along with an *increase* in the apparent variability of IPP, the uppermost-posteriormost landmark. The procedure of deflation (Algorithm II) seems to have only subtle effects on this scatterplot, in particular, tipping the apparent orientation of variation at IPP so that all of the little segmental summaries are more nearly parallel. We shall see shortly that this adjustment at IPP is serving to attenuate the single local feature manifested in these data.

To proceed further we need to examine the spectrum of the bending-energy matrix for this octagon. Figure 11 displays each of its five nontrivial eigenvectors as a grid in the orientation of the pooled growth trajectory. The specific bending energies (eigenvalues $E$ of the bending-energy matrix) are 4.3, 6.4, 14.2, 23.4, and 35.2, which is a sufficient range that the maneuver of Algorithm II should have (and does have) an effect. We see a steady drop in variance across the series of partial warps, except for the last. The question for Algorithm III is the calibration of the speed of this fall.

The plots in Fig. 12 assess this scaling. At left is the standard approach sketched in Algorithm III, the unweighted regression of log partial warp variance on log bending energy. The nominal slope here is $-1.5,$ hinting at integration rather than self-similarity, but clearly the smallest partial warp (point 5) is an outlier from the regression. We repeat the computation using only the first four partial warps. At the same time, following a suggestion of Mardia et al. (2006), we incorporate a “nugget effect” for irreducible landmark-specific digitizing noise. (This is a term for uncorrelated isotropic variance at much smaller scale than what is involved in either the self-similarity or the integrated models.) The fit is optimized for a nugget variance equal to 0.001142, which is most of the variance of partial warp 4, resulting in the considerably better fit shown in the right-hand panel together with a confirmation that partial warp 5 is somehow different.

In the presence of a hypothesis of integration, we can expect a meaningful suite of relative intrinsic warps, that is to say, relative warps of the matrix of deflated shapes (Fig. 10 lower left). An ordinary principal components analysis of these 16 Cartesian coordinates results in a first component explaining more than 91% of all the variance in the diagram, with all successive dimensions patternless according to the criterion of Bookstein (2014). It is sufficient, then, to report only this first RIW. In the deflated coordinate system it is indeed at large scale (Fig. 13, left), a combination of shortening of the upper margin relative to the lower margin with a gentle anteroposterior bending. But when we reinflate back to the original units of Procrustes distance (right panel) we see there is also a local feature at IPP, corresponding to the last partial warp in Fig. 11. Thus the growth of these skulls, strongly integrated over time (see, e.g., Bookstein 2014, Figure 7.16), is likewise strongly integrated over space, with one exception (the twist at IPP).

This pattern is strong enough that we might expect even a less sophisticated morphometric method to hint at it. The left panel of Fig. 14, for instance, confirms the presence of that single dimension of large-scale integration by an explicit principal component analysis of just the quadratic terms in this pattern of shape variation (orthonormalized terms in $$x^2,$$$$xy,$$ and $$y^2$$ for each of the two Cartesian coordinates of the deformed scene after the original $$x$$ and $$y$$ of the uniform term have been partialled out; see Bookstein 1991, Section 7.5). We see an obvious trend from the youngest rats to the oldest, with no evidence of a meaningful second dimension. This dimension is effectively the same as the ordinary first relative warp of the nonaffine shape subspace for these same data (right panel); the correlation between the two possible “factors of integration” is 0.999.

A grid diagram of this first component, Fig. 15 left, is an adequate representation of the large-scale pattern of integration beyond the uniform term. Insofar as this formula has the same second derivative everywhere, it could appropriately be characterized as “totally integrated” just as much as any uniform transformation (which has a constant first derivative) could be. If we just compute the first relative warp of the forms in the upper right panel of Fig. 10, forms that have not yet been deflated, we arrive at a grid diagram (Fig. 15 right) indistinguishable from the one at right in Fig. 13, the reinflation of the deflated analysis. This confirms the unidimensionality of Fig. 14 and hence the characterization of this rodent neural skull growth trend via four conceptually distinct processes: a uniform component that reorients itself somewhat from the first half of this growth trajectory to the second half, together with a single large-scale growth gradient, over which is superimposed a separate phenomenon local to IPP. It is comforting that two quite different kinds of geometric morphometric analysis arrive at this same finding.

Our deflation approach has thereby certainly altered the standard principal-component method, which visualizes the same sample of growth trajectories (see Figures 7.5 and 7.16 of Bookstein 2014) by just two features of shape variation that fail to attend to any aspects of scaling whether uniform, quadratic, focal, or otherwise. The first of these features is the correlated effect of the overall vertical compression (a uniform feature) with the quadratic trend and IPP detail in the complementary subspace; the second, the reversal of that direction of shear in the uniform component from the first half to the second half of the growth epoch. The method of principal components has no access to the bending energy matrix and its eigenvectors, and so cannot remark the remarkable fact that the variances of the first four partial warps, but not the fifth, are roughly the inverse squares of the ratios among the bending energies of the corresponding eigenvectors. (Certainly this finding was not reported in any of the several earlier publications on this frequently published specific data resource.) Nor can the principal-component approach focus on the salience of that single feature at the far right of Fig. 11 as the *only* feature that deviates from the smooth pattern of loglinear dropoff of partial warp variance with geometric scale. Both these findings, along with the delineation of the uniform subspace itself, are a function of the actual mean positions of the landmarks, information that the conventional multivariate analyses cannot use to interpret their covariance structure.

To be effective for reporting findings pertaining to any integrated system, a descriptive language needs to focus on the underlying parameters *of* that integration along with the feature(s) which deviate from it in just this way. Notice, in passing, that the scaling dimension identified here, $-2.2,$ does not go very far toward actually modeling the covariance structure of this data set. The value of $-2.2$ describes only the limiting slope of the plot at right in Fig. 12. Actually there are ten parameters (nine direction cosines, along with a variance) in the nonaffine relative warp of Fig. 14 (right) that exhausts the dimensionality of the nonaffine modeling here. Of those ten, six can be understood to pertain to the quadratic growth-gradient estimate, while another three, not entirely independent of the first six, specify the residual from this trend at IPP (equivalently, the residual at the fifth partial warp in Fig. 12). In any event, a count of eleven (ten plus one for the nugget variance) is far fewer than the total of 55 coefficients that would be required to notate the general covariance structure on these same 10 shape coordinate dimensions. The strength of integration in this example means that the biological context here is far from “general.”

Thus, in brief: a finding of integration is based on the estimated slope of the log-log regression of partial warp variance against bending energy, For that finding, the dominant integrated shape pattern can be visualized by the first RIW—the first relative eigenvector of the nonaffine part of a shape coordinate configuration with respect to its bending energy—and can be summarized by the corresponding quadratic growth-gradient. If a representation is desired that also visualizes the associated local modules, one might apply the same RIW formula to the undeflated partial warps instead.

### *Example 2*

The adult human callosal midcurve

In contrast to this rich feature analysis for one developing mammalian neural skull, an analysis of its contents, the mammalian brain, may show a striking *lack* of integration when restricted to (a) a single anatomical component, the corpus callosum in the midline; (b) human adult males only; and (c) a sample heavily enriched in persons with a newly discovered birth defect, fetal alcohol spectrum disorder (FASD). This 40-semilandmark, 45-subject human callosal midcurve data set was first described in Bookstein et al. (2001). It was diagrammed, and its scientific context explained, in Bookstein (2014), Figure 7.22 and the accompanying text. The sample comprises the midline curve of the corpus callosum in the brains of 15 normal Seattle adult males together with 30 adult males diagnosed with what was called either fetal alcohol syndrome (FAS) or fetal alcohol effects (FAE) at the time the sample was originally gathered. These are usually combined nowadays under the name of FASD. All diagnoses predated our study, and none involved any sort of brain imaging.

**Fig. 16:**
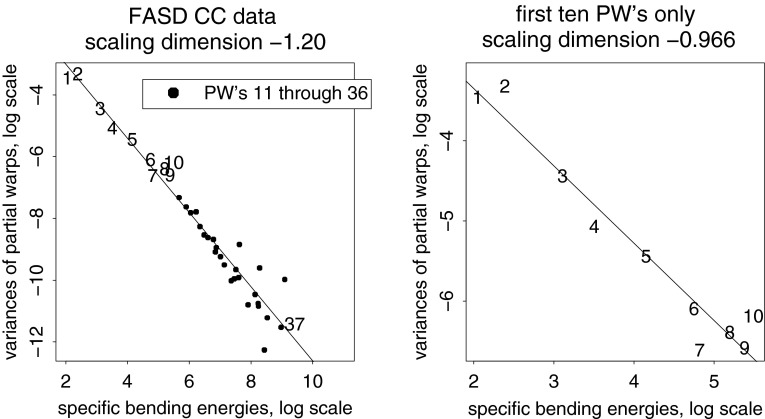
Output of Algorithm III for the callosal midcurve data. The regression slope is indistinguishable from $-1.0$ for the first ten partial warps, making moot any thought of proceeding with Algorithm IV (relative intrinsic warps), let alone ordinary principal components of shape. The anomalous ordering of the first two partial warp variances does not alter any of the interpretations in the text

The callosal outlines here were traced in three dimensions from a custom-designed brain MR protocol by a novel semilandmark procedure, the *symmetry curve,* explained in the original reference, and thereafter were projected into two dimensions for this and all earlier analyses. They are thus planar 40-gons of semilandmarks spaced roughly inversely to averaged curvature while tracing both sides of a C-shaped arc. Hence toward the high-energy (small-scale) end of its spectrum, the spacing of specific bending energies is not the discrete spectrum of Fig. 12 but the more nearly continuous spectrum of the analogous Fourier analyses. As Fig. 16 shows, in this example the scaling of log partial warp variance against log bending energy is remarkably close to the slope of $-1$ that characterizes pure self-similarity (that is, absence of spatial features at any scale), and if we concentrate on only the larger-scale aspects, the first few partial warps, the slope is almost exactly $-1.$ (The apparent nonmonotonicity for the first pair is likely an artifact of the close spacing of the first two specific bending energies.) Corrected for spatial autocorrelation, then, we are looking at precisely the spatial equivalent of Brownian motion. Any apparent large-scale features of these curves could have arisen just as well from the chance concatenation of analogously variable aspects of shape (e.g., indentations of the outline, or relative twisting of the centerline) at any or every smaller scale. These arcs no more show statistically meaningful patterns of trend than the equivalent “trends” of a random walk do (see Bookstein 2014, pp. 45–51 and 473–474). Whether the language be biopsychiatric or neuroradiological, it is not worth attempting to interpret the principal components of this shape. Properly analyzed, these outlines show no evidence of any large-scale integration. Instead, the scaling of their features is self-similar. This is not to say that the actual amplitude of their shape variation (the intercept of the linear fit in Fig. 16) is not sometimes of interest, but that the assessment of “how different” any pair of these shapes is, or what a discriminant function might look like that separates two groups of different average shapes, is going to be determined by the spacing of those (semi)landmarks in a manner that leaves the realm of biological explanation (here, the mechanisms by which alcohol interferes with the migration of glia in the embryonic brain) for the realm of medical image analysis *per se.* A finding of self-similarity, in other words, constrains the language one ought to use to *report* the shape variations within a sample—it decouples the estimates of net dissimilarity from the language of biological processes.

**Fig. 17:**
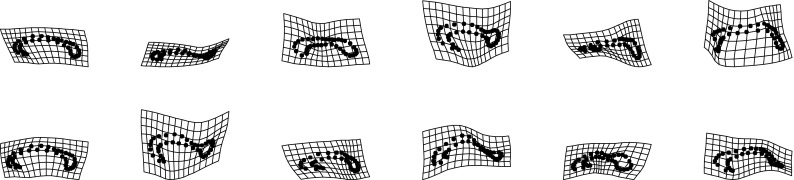
Confirmation of the self-scaling nature of the callosal midcurve data. (*above*) The first six ordinary relative warps of these 40-gons. (*below*) The same for the deflated shapes. These patterns are uninterpretable in any coherent morphogenetic context. They thereby illustrate the proposition of the text that while the ordinary principal components of Procrustes shape coordinates privilege large-scale phenomena over phenomena at smaller geometric scale (*top row*) even when applied to the description of self-similar shape distributions, the relative intrinsic warps (*bottom row*) do not do so

Figure 17 confirms this diagnosis by direct display of the first six ordinary relative warps (principal components) of the 45 outlines (above) and then the first six RIW’s of the deflated data set (below). The first ordinary relative warp (top row, left) combines a substantial component of shear with a thinning all along the arch (see Bookstein et al. 2002, Figure 7). Note that the shearing pertains to a uniform component of shape, whereas the thinning does not. This first RW is correlated with a bidirectional pattern of divergent psychometric profiles for these subjects, one in which problems with executive function are emphasized, the other emphasizing motor problems. But otherwise neither set of relative warps appears biologically suggestive when examined closely enough. In particular, each “component” of the deflated data set appears to be an arbitrary combination of features at mixed scale from all over the arch, including arch bending, height increases at one end or the other, flattening of the bulbs at one end or the other (genu or splenium), and respacings along the central segment. The haphazard mixing of all these features corresponds closely to what one would expect from independent realizations of a formally self-similar process (compare the simulations at right in Fig. 4). One might refer to the variation here as “writhing” rather than expressing any inducible morphogenetic pattern.

We make little neuroteratological progress, then, by attempting to interpret these randomly rotating mixtures as “factors of midcurve callosal shape” that might hint at stable extended patterns of spatially varying dependence on a prenatal alcohol dose. In the actual empirical scientific context generating these data, the natural history of the fetal alcohol diseases, the damage is characterized instead by the greater variance of the fetal alcohol subgroup’s outlines in the substantial majority of local shape features that might be imagined here. A suitable quantity would be analogous to the parameter for step size in the case of a random walk, or temperature or viscosity in the case of Brownian motion. Such language, while far from the biometric rhetoric of group mean differences and $t$ tests customary in neuroradiology and neuropsychiatry, nevertheless converts easily into quadratic discriminations (likelihoods based on differences of covariance matrices as well as means; see Bookstein 2014, Section 8.1) by which the shape of this outline can be used as evidence of fetal alcohol damage in adults who are encountered in the course of other societal functions. An application to mitigation in the course of the penalty phase of aggravated murder proceedings in American courtrooms is outlined in Bookstein and Kowell (2010).

I noted above that the method of deflation serves as a remedy for one nagging issue in the design of a landmark scheme, the problem of uneven spacing. We see from Example 2 that this method of deflation applies verbatim to data sets of semilandmarks without any alteration at all, and in this new context the corresponding property of being relatively independent of spacing is even more attractive. Indeed, by virtue of its intentional downweighting of local shape feature variance in proportion to the squared span of the underlying list of landmarks, the method explicitly solves the main problem that otherwise bedevils semilandmark-based morphometrics even today: how to weigh the relative information content of landmarks, curves, and (in three dimensions) surfaces for analysis of principal components and other patterns of variation. No matter how many semilandmarks there are, and whatever their spacing, the deflation method automatically reduces all but two dimensions’ worth of the full space of shape coordinates to a common weighting by inverse bending energy. One can then explore integration independent of most of these well-known pitfalls of semilandmark analysis by principal components.

### *Example 3*

Human and anthropoid skulls

For an example of potentially greater evolutionary interest I turn to a small data set of 22 *Homo sapiens* skulls and 7 others that have been analyzed before as a core example of the way a physical anthropologist ought to sequence her morphometric computations: Weber and Bookstein (2011), Chapter 4. Before that, this 22-specimen or 29-specimen data set of midsagittal cranial landmarks was described in Bookstein et al. (2003), and it is diagrammed not only in the Weber–Bookstein textbook but also in Bookstein (2014), Figure 6.8. The sample of *H. sapiens* comprises 5 human children, 16 human adults, and the Mladeč skull. There are also four Neanderthals (Atapuerca, Guattari, Petralona, and Kabwe), “Mrs. Ples” (STS 5), and two chimpanzees, one of each sex.

**Fig. 18:**
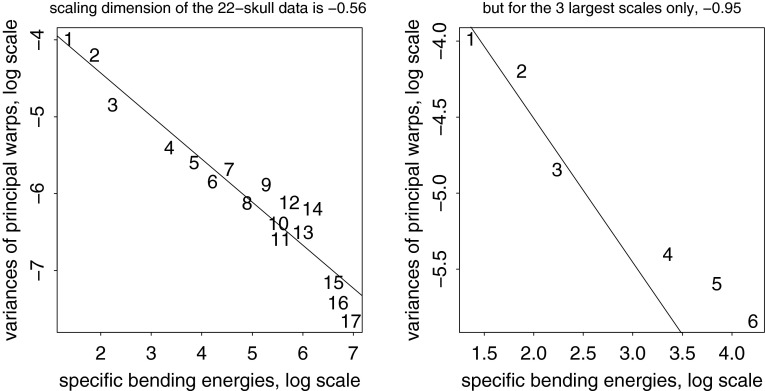
For the 22 *H. sapiens* specimens of the midsagittal skull data set, the fall of log partial warp variance with log specific bending energy is too *slow* to be consistent with self-similarity. The data set is, if anything, *disintegrated,* nonbiologically close to the totally uncorrelated Procrustes situation modelled at left in Fig. 3

As Fig. 18 shows, when the data set is restricted to the recent humans alone it not only shows no integration but actually hints at an excess of spatially uncorrelated local features—the finding called *disintegration* above—in spite of the wide age range (age 2 years through adult) that should seemingly permit any growth-gradients to have substantial leverage. The analysis here argues, in effect, that no principal components of this form in samples of any size are likely to be particularly meaningful. For instance, to arrive at a slope near the privileged value of $-1$ it is necessary to restrict the regression to an unpersuasively short list of features. (Figure 18, right, suggests this dimensionality might be as low as three.) This would correspond to a similarly short list of features remarked verbally (features characterized by terms like “globularity” or “bimaxillary protrusion”).

As in Example 2, this slope serves not only as a finding (the empirically based estimate of an informative parameter) but also as a constraint on the language of reporting—a “meta-finding,” as it were. This count of three dimensions of features is too few to be apposite to the wide range of arguments about evolutionary adaptations and functions of this structure reviewed in recent texts such as Lieberman (2011). Hence the morphometric analysis of human skull form is not likely to be informative about its biological causes or effects over samples of “typical” forms like these. The finding, in other words, is a caution about language (a caution, incidentally, that is widely ignored all across paleoanthropology).^5^

**Fig. 19:**
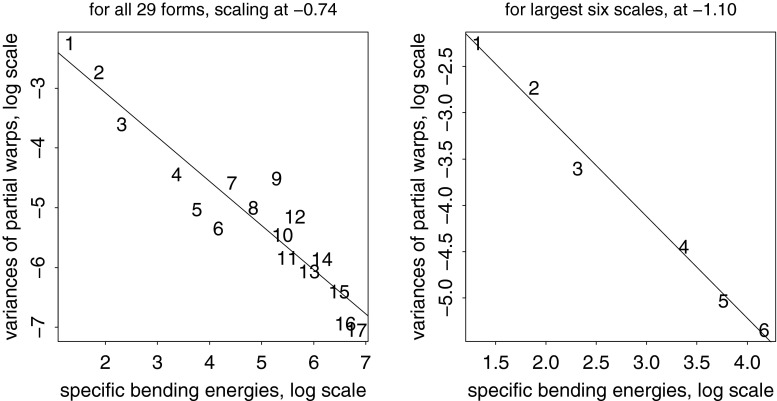
When the data set of Fig. 18 is extended by seven forms from three other genera, the possibility of a scaling dimension of $-1$ (self-similarity) appears for the range of the first few partial warps. Such a finding is consistent with models that eschew large-scale directional selection, e.g. for “neuroglobularity,” in favor of a series of fluctuations at unrelated scales

Exploring this morphospace further, we now restore the seven additional specimens (four Neanderthals, Mrs. Ples, and the male and female chimpanzees) that were part of this data set when it was originally published. In a didactic context, ordinary relative warp analysis shows that the three non-*Homo* forms do not inform us about the meaningfulness of ordinations within *Homo,* and likewise that the Neanderthals are not helpful as regards making sense of the 22 *H. sapiens* per se. A reanalysis by these methods of integration, Fig. 19, immediately confirms that judgment. A scaling dimension of $-1$ over the six highest scales cannot be argued to rule out a hypothesis of self-similar variability, meaning, in this context, that the differences between taxa and the differences within taxa, being at different morphological scales, are quite unrelated, so that principal components analysis of such a pool cannot be expected to tell us much about actual evolutionary or ontogenetic processes. Such a conclusion is consistent with a current literature that emphasizes genomic distance and other quantities consistent with models of neutral drift, especially the recent turn to such genomic analyses for more reliable information about the origins of the larger human groups (see, e.g., Pääbo 2014). In this context, so different from the domain of epigenetic explanations, the model of neutral drift in morphospace becomes more powerful as the computed slope of the log-log plots here comes closer to the value of $-1.0$ for self-similarity. In other words, neutral drift might be characterized by principal components having no particular meaning or spatiotemporal stability. It is the *absence* of meaningful principal components that renders the examination of evolutionary distances reducible to the Procrustes formulation.

**Fig. 20:**
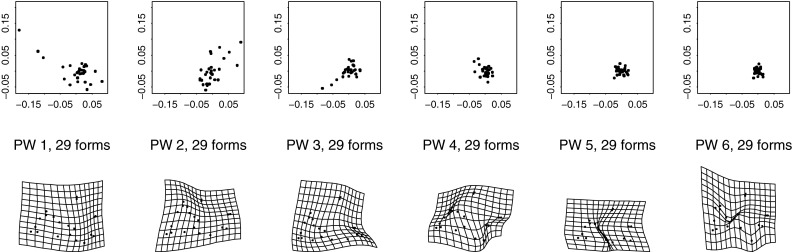
Analysis of the first six partial warp scores for the full 29-specimen data set shows a steady fall of variance with partial warp index consistent with the regressions in Fig. 19. The pattern is monotone whether or not the outlying forms (Mrs. Ples and the two chimpanzees) are considered. Such a decline is not consistent with total disintegration, but the distinction between self-similarity and integration requires the more quantitative approach of Algorithm III

The inference that samples like these afford no insights into the spatial organization of anatomical variation can be confirmed by the plots in Fig. 20, which share the design of those in Fig. 11 for the rodent skull data. We see a falloff of partial warp variance with increasing bending energy (decreasing spatial scale), but without a sharper quantitative scalpel, such as the scatterplots just proceeding, we cannot tell if this rate of decline is rapid enough to support any claim that principal components are likely to be biologically meaningful. For these six partial warps, the falloff of partial warp variance with specific bending energy is precisely in keeping with the new null hypothesis of shape self-similarity, strongly implying that examination of ordinary principal components of shape is unlikely to yield any insights. Such a prophylactic might well eliminate the majority of applications of principal components that are seen in today’s physical anthropology journals and major conferences, applications in which the extracted principal components are diagrammed as thin-plate splines without any acknowledgement of the possibility that their patterns are mainly a matter of the distribution of landmarks over the average form. It would be interesting to learn what the founders of that method, like W. W. Howells, would think about this supersession of their concerns with factor analyses, scree plots, and the like. In any event, the process of deflation can drastically alter all of the usual tabular and graphical outputs of these classical analyses. For more on the role that principal components have hitherto played in evolutionary anthropology, especially as regards functional arguments, see the discussion in Bookstein (2015a).

## Discussion

The previous section’s three examples span a realistic range of empirical possibilities, from the mildly disintegrated (the features of the 22-specimen *H. sapiens* data set, Example 3, with slope $\alpha =-0.56$) to the clearly integrated (growth of the Vilmann rodent skull octagons, $\alpha =-2.2$ for the model with a local feature and also a nugget effect). Extending this range in the direction of more integration would probably require the sort of biomechanical constraint that applies across whole extended rigid components, such as the patterns of anthropoid scapular form studied by Oxnard (1973).

I was not able to locate data that generated a value of this slope $\alpha$ any closer than −0.56 to zero, its value for the isotropic offset Gaussian shape distributions that drive most textbook presentations today. Greater extents of disintegration probably require contexts with a substantial component of sheer digitizing error, such as the isotropic offset Gaussian distribution itself (Fig. 3, upper right). It would appear that the case of $\alpha =0$ is thus not suitable as a biological null hypothesis, since it is practically never encountered in real data sets. The provenance of a realistic null does not appear to characterize any of the currently popular approaches to “testing” integration. Among the candidates that do not meet this very reasonable criterion are Mantel tests comparing empirical distance or dissimilarity matrices to simplified models, and permutation tests of landmark rearrangements that do not correspond to noise processes having anything to do with the factors known to oversee organismal shape variation. But this manuscript is not the place for a sustained critique of that other literature.

Even a cursory glance at the shapes in Fig. 4 that arise from the isotropic offset Gaussian shows their irrelevance for organismal biological questions. If landmark perturbations from a mean form were indeed independent from landmark to landmark, the form you are studying would not have been regulated; it (your ancestor, if the study is about the evolution of *H. sapiens*) would have died before growing into that configuration. (Thus you would not be encountered in the fossil record, either.) Although the offset isotropic Gaussian for landmarks in two- or three-dimensional space (the archetypical disintegrated distribution) is the mathematical equivalent of stasis in time, it is not the scientific equivalent—owing to the way that organismal development is actually regulated, it is never seen, and so is not a process of any interest to the organismal biologist. Then, obviously, it cannot function as a null model should—it has no chance of serving as a meaningful analysis of any situation involving living organisms. It is pointless to report the rejection of hypotheses of pure noise when we are actually interested in delineating the factors accounting for correlations of landmark locations from region to region across an entire organismal form. The symmetries of that pure noise model are so distant from the factors of any real organismal course as to be irrelevant to their visualization or explanation.

Hence the totally disintegrated models found in the current morphometric literature seem far less congenial to biological explanations than the self-similar model introduced here. The hypothesis of self-similarity, in fact, has been shown to be consistent with data from two of the three exemplary data sets reviewed above. By contrast, when integration proves to be present, as in the rodent skull example, our purpose is to *describe* its features, at whatever scales they are manifested. The appropriate comparison for the growth gradient unearthed in the rodent data is not against the null of isotropy, but against the much more apposite null of self-similarity, and, when even that subtler null is found not to fit, the further comparison of the variance on partial warp 5 with the scaling dimension of the preceding four partial warps, the comparison that confirms the local effect at IPP. In this data set, both of the comparisons meet the “interocular trauma test” (Bookstein 2014)—both “hit you between the eyes.” There is no need for any further statistical computations. Rather, the biologist, dismissing the morphometrician with thanks, can proceed straight to the stage of a biometric interpretation, the way Vilmann and Moss always wanted to go.

**Fig. 21:**
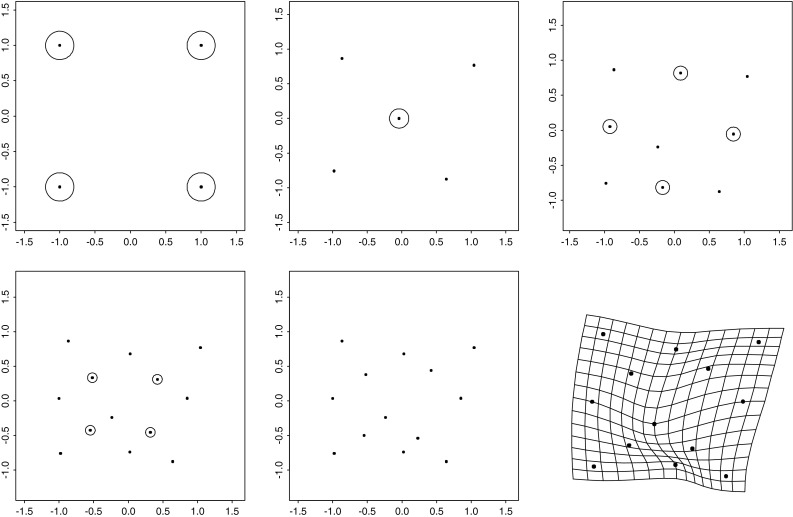
For templates that are close to grids in their spacing, approximately deflated deformations may be constructed serially from a parcellation into cells involving one new landmark each with isotropic variance that is linearly scaled to the area of its compartment

Approximations are available that replace the formal construction of the self-similar distributions (as in Fig. 4) with more graphical versions easier to teach and digest. One such explicit generative model, originally published in Bookstein (2007), is diagrammed in Fig. 21. A 13-landmark template reminiscent of graph paper is parcellated into successively smaller compartments within each of which the variability is represented by one “new” landmark varying with circular symmetry at a variance that shrinks with the size of its compartment. Regarding the prototype in Fig. 21, for instance, the first four landmarks are the outer corners of the square that jointly delimit a Procrustes shape space of the four dimensions shown in the middle row of Fig. 8 (Bookstein 1991). The fifth landmark, at the center of the square, is perturbed with circular symmetry around the location imputed to it by the deformation of the square, with a variance that is half that of the corners of the square. Then the midpoints of the edges of the square follow, independently in this simulation, each perturbed around *its* imputed location with variance reduced by a further factor of one-half, and so forth. If we stop at the 13-landmark stage, lower right, the resulting net deformation (graphed of course as a thin-plate spline) appears to have discrete features at a satisfying range of spatial scales. If this were a summary of some experimental or evolutionary phenomenon, we would be able to report it and speculate on its causes or effects using a language of a hierarchy of scales rather as we did for the real example of the rodent skull data. (If the goal were to simulate the rodent data in particular, the variance would drop faster than the areas of the cells, and there would be some directional information injected, too.) When the mean landmark positions involve such artificial symmetries, parcellations like this can be extended indefinitely, and you can see how they are self-similar, or nearly, by explicit design.

**Fig. 22:**
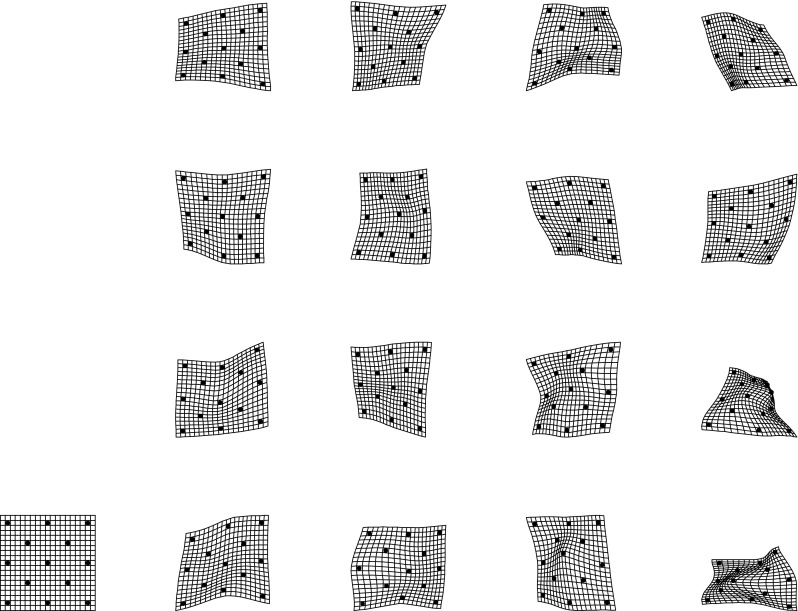
Examples of these simulations circumvent all of the obvious artificialities of the equivalent Procrustes (isotropic) distributions. They are much more likely to sustain a short list of biologically comprehensible features consistent with evolutionary or developmental explanations. All are both integrated and modular by explicit construction; those notions are not opposites in any morphometrically useful sense. Far left: the starting form (a regular grid). Center left to right: samples of these deformations along a steadily increasing list of self-similar amplitudes

Figure 22 shows a sampling of forms produced from multiple runs of this hierarchical procedure for different settings of the amplitude of the initial perturbations (along with all that follow). Now each deformation of the template seems to suggest a short list of one or two specific features *of* that deformation, a circumstance entirely contrary to that of the analogous offset isotropic shape distribution (recall Fig. 4). Taken as a whole, the models of a sample such as this are (nearly) self-similar in the sense used here. But as samples of 1 they have features that could well be worth reporting, as for purposes of classification or medical diagnosis. In other words, if you encountered just *one* of these grids in real data (or anywhere else outside the context of this specific simulation) the issue would not be to test it “against a null of isotropy,” because isotropy is not a tenable theory of morphogenesis. Instead the task would be to *describe* its features—what gradient(s) it bears, and at what spatial scale(s). That these descriptors might prove unstable in larger samples should not vitiate their relevance for the description of the individual grid.

What about the uniform term? Prior to deflation we sequestered the uniform dimension of shape variation, a total of two degrees of freedom, on the grounds that it has zero bending energy regardless of its amplitude, and thus could not be made commensurate with the rest of the Procrustes shape space in terms of an inverse bending energy. Of course, that uniform term has a magnitude of its own derived from the original geometry of the Procrustes tangent space, a magnitude that derives from the two-dimensional projection onto this subspace already set out in Eq. (7). One could reasonably wish that those last two (or, rather, first two) degrees of freedom ought to be incorporated somehow in plots like those here. And they can be, as long as it is understood that, like point 5 on the right side of Fig. 12, they have been omitted from the computation of the scaling dimension. Specifically, one can plot a point corresponding to “partial warp 0” on the other end of the abscissa of analyses like these, and use the Procrustes amplitude of the corresponding term to (perhaps) shift the upper limit of the ordinate in this same plot. Its exact abscissa is indeterminate, of course. If there is a linear fit with slope steeper than $-1,$ one might locate it at a fictive bending energy corresponding to the amplitude of this uniform variation, and interpret it as the “scale” of the corresponding “bending.” For these Vilmann data, the uniform term has variance 1.74 times that of partial warp 1, and so would be plotted $(\log 1.74)/2.2\sim 0.252$ to the left of the point labelled 1 in Fig. 12 (right), where it would appear likewise to lie fairly close to the nugget-modified regression line.

It would be tempting to presume that for data sets with high negative scaling dimension, like the Vilmann rodent neurocrania, one ought to expect a high correlation between the first relative intrinsic warp and this uniform term. But this Vilmann example actually rebuts that expectation. As the text noted, the uniform term has two dimensions, one of which reverses over developmental time, even though the nonaffine term has only one dimension, which does not reverse. In terms of our ad-hoc modification of Fig. 12, this means that the point corresponding to the *vertical compression* of the uniform term stands for only one dimension, not two, and so should be plotted twice as far to the left of the point for partial warp 1 as we just indicated. In this position it falls well below the fitted curve in its vicinity. That is because, as we have seen, only one of its components appears to be integrated with the nonaffine part of shape, not two. The relation between a dimension of strong nonaffine integration and the two dimensions of uniform transformation is thus a matter of empirical investigation, not theorems. For data that resemble our other two examples—the self-similarity of the callosal midcurves, or the mild disintegration of the human skulls—there is no finding of integration to be extended to this uniform subspace, and thus no pooled analysis to be put forward.

The dependence of the isotropic Mardia–Dryden model for Procrustes shape coordinate covariances (Figs. 1 or 3) upon the mean Procrustes configuration has not gone unremarked in earlier critiques of geometric morphometrics. The explicit effect of a small change in mean shape on the “totally disintegrated” covariance structure was set out as a relative eigenanalysis in Bookstein (2009), but the resulting “corrections” still fail to take realistic spatial autocorrelations into account. These hitherto-unformalized features of all known organismal data sets have been a particular concern of Philipp Mitteroecker in several recent articles, e.g., Mitteroecker (2009) or Mitteroecker et al. (2012). When irregularities of landmark spacing are of particular concern or when the distribution of semilandmarks is incommensurate with that of the proper landmarks, Huttegger and Mitteroecker (2011) suggested that the corresponding descriptors should be limited to those that were “affinely invariant,” that is, techniques like relative eigenanalysis that are robust against uniform changes of the parameter space. Notice that, according to Eq. (7), the Procrustes mean form is present *explicitly* in the uniform term of Procrustes shape space via both its actual coordinates (the coefficients $x_i$ and $y_i$ there, which vary from form to form) and its principal moments $\alpha$ and $\gamma ,$ which are functions only of the mean form. The present paper’s suggestion that that variation be sequestered for separate treatment might prove a more satisfactory compromise in practice. In a context of positive covariance matrices, such as those arising from interlandmark distances in the presence of a dominant size factor, the need for a method that permitted simultaneous findings at multiple scales (e.g., integration along with modularity) was already set out as a desideratum of a modified geometric morphometric toolkit in Mitteroecker and Bookstein (2007). The deflation technique in this paper might speak to all of these concerns of Mitteroecker’s except, intriguingly, the concern for phenomena at the very largest scale (the scale of zero bending energy), the uniform term itself.

The model of self-similarity, as it separates the domains of integration and disintegration, aligns with several distinctions that the reader may have encountered before. The overall growth gradient and the single feature local to IPP that characterize the rodent data set relate to the self-similar models as directional drift and punctuation, respectively, relate to the neutral drift models that exemplify classical random walk. Just as time series having a trend show variance over time that grows faster than linearly with time interval, so do growth-gradients show variances of partial warps that grow more rapidly than the reciprocal of bending energy over an analogous range of spatial scales. We have thus arrived at an analysis of spatiotemporal phenomena that generalizes the existing toolkit of purely temporal processes whenever the spatial domain can be represented by discrete landmark configurations in the usual morphometric way. We can even imagine a cross-classification of the spatial by the temporal, with joint models (or findings) that might announce a static self-similarity, a trend in the parameter of disintegration, or (the form likeliest to be of interest in evo-devo studies) a temporal trend or phylogenetic pattern for features that are found to be integrated already, like the combination of a growth-gradient with a local parietal rearrangement that characterized Vilmann’s rodent skulls.

The self-similar transformations are likewise a close spatial analogue of the Felsenstein (2004) version of phylogenetically independent contrasts once they are scaled to unit time interval by dividing by the square root of divergence time. The formal reason for the division is the same in both domains: to test the plausibility of a model of putatively independent dimensions all having the same variance, so that the pooled covariance structure ought to be spherical. In the isotropic Procrustes model, the shape coordinates themselves are distributed spherically within their subspace, but that is not the subspace of biologically meaningful feature extractions. Similarly, in the Felsenstein approach, it is not the species means per se that comprise the substrate of independent identically distributed samples, but the contrasts, which are the analogue of our deflated partial warps here. Thus “it has not escaped our notice,” as the sly trope goes, that the entire machinery of deflation introduced here via a presumption of independent samples of specimens or rodent neurocranial growth trajectories could be translated unchanged into a context of phylogenetic inference. A follow-up manuscript on this theme is currently in progress.

Another contextualization of this multiscale approach is as a generalization of what we already do with bilateral asymmetry by following the protocol of Mardia et al. 2000 for landmark data. This treatment rotates the entire descriptor space from the a-priori Procrustes basis to a considerably altered one explicitly incorporating the biologist’s prior knowledge of which landmarks are unpaired, which paired, and, for the paired landmarks, which are on the left and which on the right. The expectation is that the variance of the side-to-side contrasts in this new basis will be much less than the variance of their original and mirrored averages, justifying the separate reports of a “symmetrization” together with terms for fluctuating and directional asymmetry to which we have become accustomed over the last several years. The model is thus a truncated version of the model of self-similarity here, with only two subspaces instead of arbitrarily many and usually with only one parameter (FA, fluctuating asymmetry, treated as a scalar sum of squares) for modeling variance in a spherical (directionless) way within that subspace of smaller variance.

That bilateral symmetry can be imagined a discretization of a spatial scaling analysis may be a variant of the effectiveness, in many studies of dynamical systems, of segregating its responses to perturbations into two distinctive domains, a “fast” and a “slow,” differing substantially in their temporal characteristics. The appeal of principal components for studies of evolutionary trends presumes, in effect, that the large-scale features of spatial configuration are also those of the largest scale in evolutionary time—the ones for which change is easiest to explain in terms of selection. But, as Charles Oxnard (1967) noted a long time ago, the aspects of shape associated with function and the aspects associated with longterm taxonomic change are more often orthogonal than parallel. There is an extended discussion of the relation between principal components and functional morphology in Bookstein (2015a).

Beyond morphometrics, any parameterization that makes explicit the spatial scaling of descriptive models may articulate to diverse other current themes of statistical data analysis in the natural sciences. Centering a null model nearer to the typical data set certainly adds both statistical and descriptive power in a great range of contexts. My examples entailed an unusual version of this strategy in which a subclass of covariance matrices was highlighted as an explicit function of the mean shape even though that mean itself was estimated in the usual fashion. The null of self-similarity matches the typical assignment of shape feature extraction in the same way that the analogous null model of independent increments sharpens the analysis of trends into the study of autoregressive processes. By invoking self-similarity one can circumvent the otherwise daunting truism that every physical instrument (in our case, every imaging device) has a finite aperture of signal sensitivity (Koenderink 1990). Whenever one of the log-log plots exemplified here proves to have a well-characterized slope, one can explicitly model the expected effect of a change in that aperture. This might well be a useful insight into a variety of current extensions of brain imaging into the realms of smaller spatial scale—for image types such as diffusion tensors or the “connectome,” a probability model confirmable at larger spatial scales might support a useful extrapolation downward to smaller spatial scales even in the absence of actual microdata on the same specimens. Other domains would privilege other null construals of a scaling dimension. Biomechanical properties (such as strain) often vary as a different scaling of form (see the sketches in Bookstein 2013a), and scaling analyses of trabecular bone translate into the arithmetic of finite strain analysis under the heading of homogeneity studies. This sort of modeling is also reminiscent of the *power laws* we use to assess and then normalize scaling effects in studies that range from branching structures of watersheds, through bronchial or vascular trees, to social media and the Internet.

More abstractly, one generally salient principle instantiated in this essay may be the way it conflates a parametric modeling task (the subspace of covariance matrices that are in fact self-similar for a particular mean shape) with a correspondingly disciplined mode of discourse (the rejection of all claims that individual features have been identified except insofar as they deviate from that implied regression). For the interpretation of a covariance structure to depend explicitly on the mean form however independently estimated is certainly an uncommon aspect of Gaussian modeling strategies; for the analysis of that same covariance structure to have a null that is not reducible to any easily parameterized subspace of the Wishart space is equally atypical. Other fields might well have equivalents of this dependency, for instance, the way invoking a technique quite similar to kriging in environmetrics allows one to predict the expected density of air pollutants at locations in-between the ones sampled, in a manner that greatly rewards care in the spatial relationships of the sampling locations themselves (Cressie and Wikle 2011), or the way that interpretations of intelligence or achievement test scores depend partly on the prior knowledge of the item pools from which the actual test items were drawn (Lord and Novick 1968). For such choices to have empirical consequences, there needs to be a formalism for the space of possible measurement vectors just as much as a formalism for the design of the samples of specimens considered; and those measurement vectors may have a representation that echoes our approach to landmark locations and spacing. In other words: where geometric morphometrics shows its sturdiest ties with biology is in the understanding of how landmark locations can arise from properties of the growing or functioning organism and how the phrasing of those connections depends on details of the landmark schemes driving the explanations.

Yet extensions and intellectual analogies of this flavor are more speculative than the explicit morphometric examples put forward in this essay. It is my hope that the publication of the initial algorithm here will launch an injection of explicit geometrical modeling into a current toolkit for integration studies that is seriously lacking in tools competent to handle most contemporary morphometric hypotheses. We need methods that exploit the explicit quantitative geometry of the observed mean configuration of landmarks so as to condition our interpretation of their covariance structure on their spacing. Furthermore, in my judgment, the trichotomy of integration–self-similarity–disintegration should replace the currently fashionable polarity of integration “versus” modularity. Those two terms do not lie in the proper biometric relationship to serve as opposite polarities in this way—no hypothesis lies between them, and both can apply in the same data set. (The pattern of the Vilmann data is obviously a long way into the integrated regime, and yet it clearly shows patterns at two scales, local and global. This data example is thereby both integrated *and* modular.) An analogous transition occurred in multivariate statistics nearly ninety years ago when the field was stimulated to move from the Gaussian (ellipsoidal) model for joint distributions of *data vectors* to John Wishart’s celebrated model of 1928 for the higher-dimensional distribution of the *covariance matrices* that summarize those same multivariate Gaussian vectors. From this transition arose most of today’s language for assessing the sampling variability of principal components and factors in otherwise unstructured variable sets. See, in general, Bookstein (2015b), Chapter 4. We are still building on that Wishart foundation today. But the suites of variables we exploit in morphometrics are no longer unstructured lists, and that additional (spatial) structure changes everything.

For studies where each variable arrives with some accompanying spatial information, the way geometric morphometrics exploits the vector of all the shape coordinate means, we need likewise an operation that breaks the nonbiological symmetries of Procrustes shape space in favor of a scheme explicitly incorporating the mean landmark configuration as part of its algebra. The approach here, specifically, its deflation step, meets that requirement. (I am not claiming that it is unique. Other approaches to multiscale analysis of anatomical images have been explored, for instance, Seiler’s (2012) hierarchical subdivision of the human mandible, that may, after suitable modifications, be applicable to landmark data as well.) The Wishart model required new mathematics, or, rather, mathematics imported from other branches of science than morphometrics. The enrichment I am suggesting here likewise required new mathematics, in this case, the reinterpretation of the properties of the thin-plate spline set out by Kent and Mardia in their great paper on kriging. Corresponding to this algebraic transition there needs to be a family of descriptive terms matching the contrasts characterizing the actual empirical context in which questions arise. This paper suggests that such a context should be the apparent amplitude (variance) of shape features as a function of geometric scale, divided into two unbounded regimes (integration or disintegration) separated by the dividing submanifold of self-similarity, which is a proper point hypothesis as regards the regression slope that is being estimated. The self-similar models are the appropriate null models here just as the low-dimensional models (such as the single-factor models) are the appropriate null models for covariance matrix studies. But there is far more information available in landmark-based morphometrics than mere covariances. Whenever patterns of shape change are different from landmark to landmark or from direction to direction, the methods that refer only to covariance structures fail to lead to appropriate biological insights.

In short, morphometrics is not just a matter of interspecimen distances, whether according to the Procrustes formula or any other. Far more information is encoded in our conventional landmark data structures than what is tapped by the conventional toolkit of Procrustes shape coordinates. It is high time that the information from the mean shape be made accessible to the pattern analysis of how shape coordinates vary around their mean and the biological implications of these patterns for growth, form, or evolution. The technique of deflation introduced here is one such explicit invocation of the mean shape for purposes of organizing the further morphometric analysis of shape spaces, and surely there will be further contributions along these lines in the years to come. The great philosopher of science Karl Popper referred to the two principal themes of natural science, determinacy and indeterminacy, as “clocks and clouds.” In a famous lecture published as Popper (1966) he pointed out the intentionally sly mendacity of this dichotomy: all clocks are clouds, all clouds are clocks. The applied mathematics by which the information in clouds is to be parameterized as covariances requires principles of scaling not only for “clocks,” the numéraire of evolutionary time, but also for “clouds,” the atlases that authorize us to oversee the mensuration of space. An extension of morphometric scaling from the temporal to the spatial domain will very likely accelerate all the ways we retrieve information about biological process from the extended organismal images that supply our primary phenetic data resource.

## Footnotes

1 This term is intentionally written without the hyphen: “disintegrated” instead of “dis-integrated.” Likewise the noun in this paper’s title is “disintegration” instead of “dis-integration.” The intention is to preserve not only the pronunciation (elision of the $s$ with the short $i$ that follows it) but the dynamic connotations of the term in existing contexts where it is always unhyphenated. There is an aggressively nonbiological character to the deformation grids in the left panel of Fig. 4. It is difficult to imagine any biomathematically plausible differential equation, morphogenetic or otherwise, that would be capable of producing results so disorganized.

2 The data sets to which I have access offer no examples of completely disintegrated distributions. This model appears to be an option characteristic of textbook examples rather than systems describing the world of living or previously living metazoa.

3 This odd term, pronounced “kreeg-ing,” derives from the name of its originator, the South African mining engineer D. G. Krige, 1919–2013. Krige’s gifted empirical work of the 1950’s was formalized for mathematicians by the probabilist Georges Matheron in subsequent decades, and now forms the foundation of the field known as geostatistics (see, e.g., Cressie 1991).

4 This construction is analogous to the classical definition of Gaussian curvature as the limit as path diameter approaches zero of the squared failure of a square path to close on a curving surface, divided by the area of the square. In effect we are assessing the variance of an estimated curvature.

5 For a data set to be considered disintegrated according to the classification here, the variances of warps at the largest available spatial scales must fall short of what the null hypothesis of selfsimilarity would assign to them given the variances of the patterns at smaller spatial scales. Thus an alternative way of phrasing the finding is to note that the largest-scale patterns of shape variation seem to be canalized. Even the first principal component of a data set could accommodate this description. Maximizing variance as a multiple of the squared length of a vector of loadings in Procrustes shape space is no guarantee of morphogenetic or phylogenetic meaning, any more than the first principal component of a vector random walk in time has to be meaningful (Bookstein 2013)—the data set must first be found to be integrated.
